# The Isoenzymic Diketocamphane Monooxygenases of *Pseudomonas putida* ATCC 17453—An Episodic History and Still Mysterious after 60 Years

**DOI:** 10.3390/microorganisms9122593

**Published:** 2021-12-15

**Authors:** Andrew Willetts

**Affiliations:** Curnow Consultancies, Helston TR13 9PQ, UK; andrewj.willetts@btconnect.com; Tel.: +44-7966-968487; Fax: +44-1392-851304

**Keywords:** ketolactonase, diketocamphane monooxygenase, flavin-dependent two-component monooxygenases, ferric-flavin reductase, putidaredoxin reductase

## Abstract

Researching the involvement of molecular oxygen in the degradation of the naturally occurring bicyclic terpene camphor has generated a six-decade history of fascinating monooxygenase biochemistry. While an extensive bibliography exists reporting the many varied studies on camphor 5-monooxygenase, the initiating enzyme of the relevant catabolic pathway in *Pseudomonas putida* ATCC 17453, the equivalent recorded history of the isoenzymic diketocamphane monooxygenases, the enzymes that facilitate the initial ring cleavage of the bicyclic terpene, is both less extensive and more enigmatic. First referred to as ‘ketolactonase—an enzyme for cyclic lactonization’—the enzyme now classified as 2,5-diketocamphane 1,2-monooxygenase (EC 1.14.14.108) holds a special place in the history of oxygen-dependent biochemistry, being the first biocatalyst confirmed to undertake a biooxygenation reaction equivalent to the peracid-catalysed Baeyer–Villiger chemical oxidation first reported in the late 19th century. However, following that auspicious beginning, the biochemistry of EC 1.14.14.108, and its isoenzymic partner 3,6-diketocamphane 1,6-monooxygenase (EC 1.14.14.155) was dogged for many years by the mistaken belief that the enzymes were true flavoproteins that function with a tightly-bound flavin cofactor in the active site. This misconception led to a number of erroneous interpretations of relevant experimental data. It is only in the last decade, initially as the result of pure serendipity, that these enzymes have been confirmed to be members of a relatively recently discovered class of oxygen-dependent enzymes, the flavin-dependent two-component monooxygenases. This has promoted a renaissance of interest in the enzymes, resulting in programmes of research that have significantly expanded current knowledge of both their mode of action and regulation in camphor-grown *P. putida* ATCC 17453. However, some features of the biochemistry of the isoenzymic diketocamphane monooxygenases remain currently unexplained. It is the episodic history of these enzymes and some of what remains unresolved that are the principal subjects of this review.

## 1. Introduction

**Figure 1:**
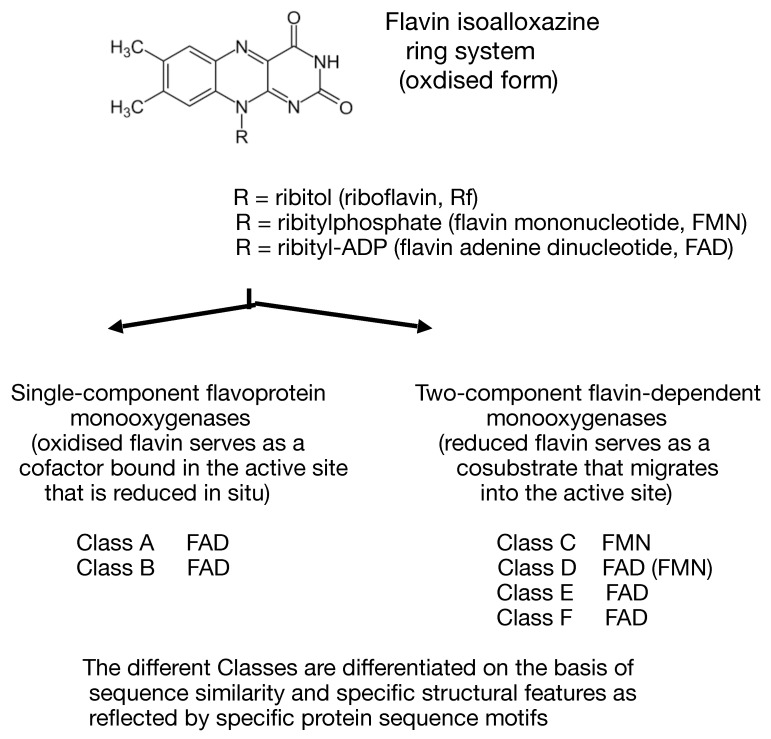
Structure of the flavins and the major enzyme classes they support.

Monooxygenases that deploy one or more flavin moieties (riboflavin [Rf], flavin mononucleotide [FMN], and flavin adenine dinucleotide [FAD]) as an integral contributor to their mode of action can be subdivided into two major groups (Figure 1, [1]). Single-component flavoprotein monooxygenases, which have been studied for several decades, are true flavoprotein enzymes that contain one or more tightly-bound oxidised flavin moieties serving as cofactors in the active site which are directly reduced in situ by NAD(P)H sourced from one or more separate complementary enzymes. Conversely, the functionally distinct flavin-dependent two-component monooxygenases (fd-TCMOs, [2]) are an emerging group of enzymes that are not true flavoproteins as they contain no bound flavin, but rather deploy a reduced flavin as a preformed cosubstrate sourced externally either by a sequential or ping-pong reaction mechanism [3] from one or more separate NAD(P)H;flavin oxidoreductases [4].

**Figure 2:**
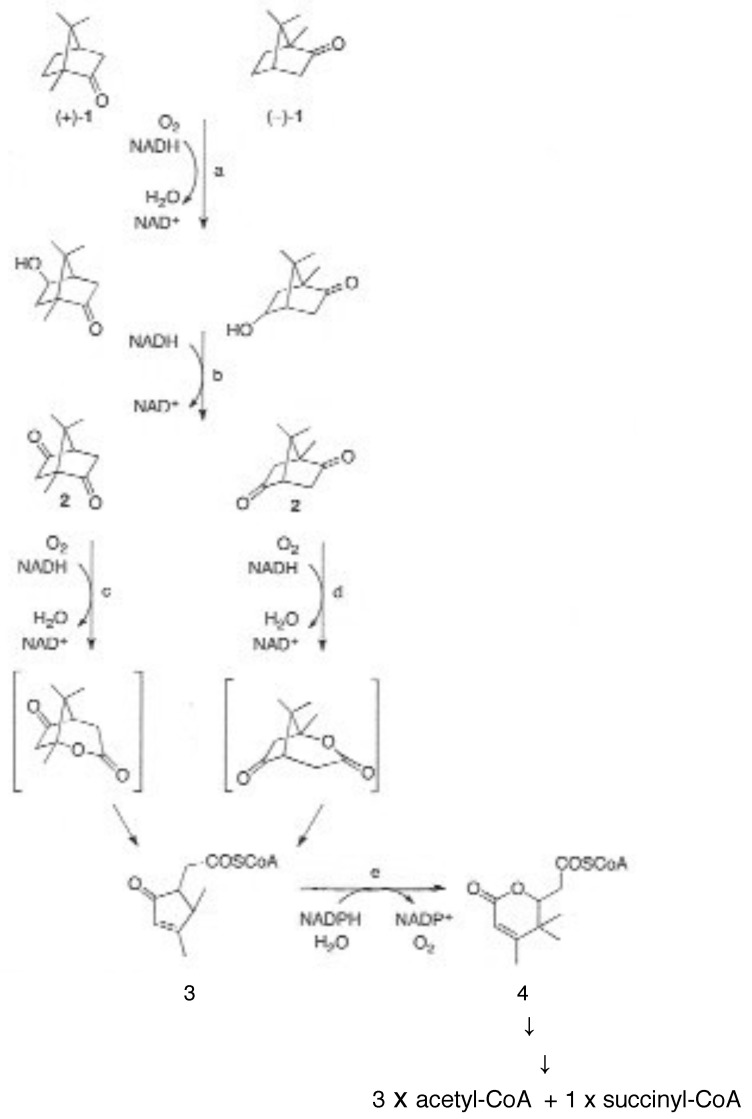
Dedicated pathway for the degradation of (+)- and (-)-camphor (1) by *Pseudomonas putida* ATCC 17453. Key idiosyncratic pathway intermediates shown are the enantiomeric diketocamphanes (2, DKCs), the monocyclic ketone 2-oxo-△^3,4^-4,5,5-trimethylcyclopentenylacetyl-CoA (3, HTP-CoA) itself formed from the corresponding transitory free acid precursor OTE (2-oxo-△^3,4^-4,5,5-trimethylcyclopentenylacetic acid), and the unstable monocyclic lactone 5-hydroxy-3,4,4-trimethyl-△^2^-pimelyl-**δ**-CoA lactone (4). Subsequent hydrolysis of the monocyclic lactone generates the first aliphatic intermediate, a C10 carboxylic acid that is further metabolised to the TCA cycle intermediates acetyl-CoA and succinyl-CoA. The key sequential enzyme-catalysed steps are: a. camphor 5-monooxygenase (*camCAB*); b. *exo*hydroxycamphor dehydrogenase (*camD*); c. 2,5-diketocamphane 1,2-monooxygenase (*camE_25-1_* + *camE_25-2_*); d. 3,6-diketocamphane 1,6-monooxygenase (*camE_36_*); e. 2-oxo-△^3,4^-4,5,5-trimethylcyclopentenylacetyl-CoA monooxygenase (*camG*).

The first flavin-dependent enzyme to be studied in any detail was in fact not a monooxygenase, but an oxidoreductase isolated from the bottom fermenting yeast *Saccharomyces carlsbergensis* that was convincingly confirmed to be a true flavoprotein in the early 1930s [5]. Because of that historical significance, the enzyme subsequently became widely referred to as Old Yellow Enzyme (OYE, [6]), yet more than 80 years after it was first studied, aspects of the biochemistry and functionality of that originally discovered true flavoprotein and its homologues remain enigmatic [7]. Similarly, 2,5- and 3,6-diketocamphane monooxygenase (2,5- and 3,6-DKCMO), which are isoenzymic Class C fd-TCMOs coded for by corresponding genes located on the large 533 kb CAM plasmid of *Pseudomonas putida* ATCC 17453 [8], are also characterised by an equivalent chequered history that spans almost six decades. These enzymes, which play key enantiocomplementary enabling roles in the degradation by the bacterium of the two isomeric forms of the naturally occurring C10 bicyclic terpene camphor (Figure 2), were first studied in the early 1960s [9]. The protracted emergence of our fuller current understanding of both the form and function of the DKCMOs has been influenced by a cocktail of contributory factors, some positive (including the evolution of more relevant concepts and ideas, plus the development of more refined techniques, allied to more powerful instrumentation), some negative (including misinterpretation, both of de novo data and extant literature) and, perversely, some of the most important by pure serendipity. The catalogue of relevant events represents not only a paradigm of the way science in general progresses (evidenced typically by the discovery of the true structure of DNA [10]), but also serves to emphasise the idiosyncratic episodic history that has characterised the scientific journey from their initial discovery to our current understanding of the isoenzymic DKCMOs of camphor-grown *P. putida* ATCC 17453. It is the fascinating and still evolving history of these enantiocomplementary enzymes, and the generic lessons that can be learnt from it, that are the major foci of this review.

## 2. The Oxygen ‘Revolution’ and the Origins of the Monooxygenases

**Figure 3:**
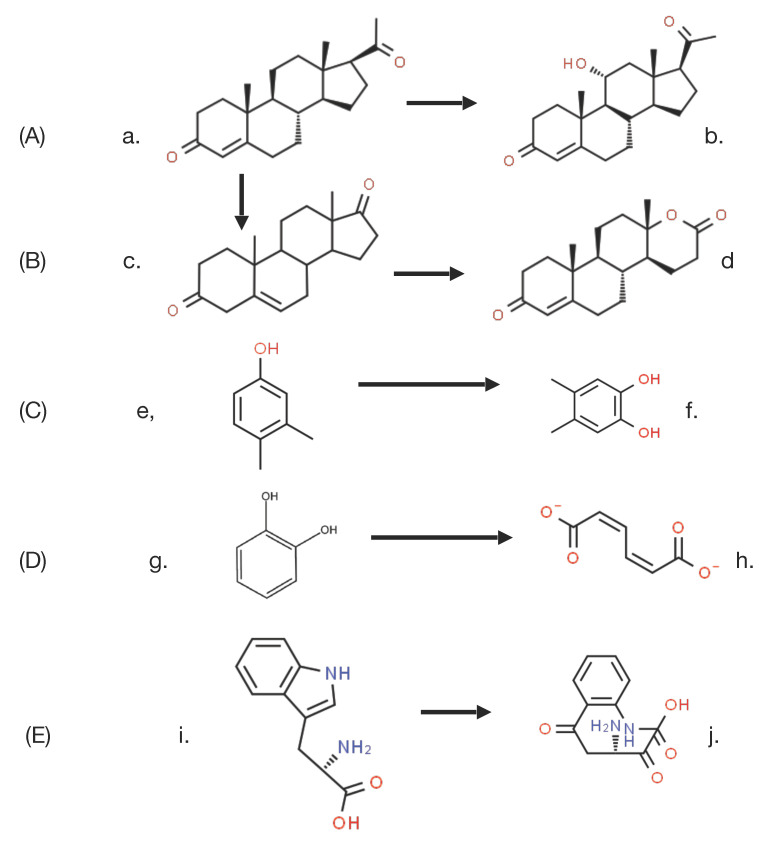
(**A**–**E**) Examples of the earliest confirmed O_2_-dependent enzymes assayed in either whole-cell or cell-free systems. a. progesterone; b. 11⍺-hydroxyprogesterone; c. 4-androstene-3,17-dione; d. testololactone; e. 3,4-dimethylphenol; f. 4,6-dimethyl-catechol; g. catechol; h. cis,cis-muconate; i. tryptophan; j. N-formylkynurenine.

The key driver that set in train the events that led to the discovery of the enantiocomplementary DKCMOs in camphor-grown *P. putida* ATCC 17453 was the commencement in the mid 1950s of research both in the US and Japan to challenge the then accepted wisdom to explain biological oxygenation processes. In the several decades that preceded the 1950s, the dominant influence in investigating all oxidation processes in living cells was Professor Heinrich Weiland of Munich University. As a Nobel laureate in chemistry (1927), his views were all pervasive, and his consistent belief, first promulgated in the 1930s [11], was that oxygenated metabolites resulted exclusively from biochemical reactions dependent on sourcing oxygen atoms from molecules of water. As such, he perceived no equivalent functional role for diatomic oxygen. At the time, the number of such reactions that were recognised was relatively small, but did include a number of ring-opening biotransformations such as the cleavage of the heterocyclic ring of tryptophan to N-formylkynurenine by liver microsomal preparations [12], and the cleavage of the A-ring of cholesterol to Windans’s keto acid by a *Protoactinomyces* sp. [13]. Additionally recognised was the hydroxylation of cholesterol to an incompletely characterised product by whole cell cultures of an *Azotobacter* sp. [14]. These and other equivalent biotranformations attracted little contemporaneous interest as they were considered to be essentially irrelevant biochemical novelties devoid of any commercial significance. However, that perception changed very significantly towards the end of the 1940s, initially as a result of the recognition by the pharmaceutical industry of the therapeutic value of some regioselectively hydroxylated steroids, such as the anti-inflammatory effect of C11α-hydroxy steroids. This precipitated a flurry of commercially-driven activity which soon resulted in the subsequent discovery in the early 1950s of the ability of whole cell cultures of fungi such as *Aspergillus niger* [15] and *Rhizopus oryzae* [16] to undertake the 11α-regioselective biohydroxylation of progesterone (Figure 3A), this being a reaction that could only be achieved by low-yielding multi-step conventional chemical means. This initial success was quickly followed by the characterisation of the step-wise biotransformation of progesterone via 4-androstene-3,17-dione to testololactone (Figure 3B) by whole-cell cultures of various fungi and bacteria [17,18], a reaction sequence that involved two successive biooxygenations, including D-ring expansion of the diketone to generate the equivalent δ-valerolactone. These and a number of other equivalent discoveries quickly progressed into a growing awareness of the necessity to understand, and thereby hopefully be able to control and ultimately optimise, the enzymology responsible for such biooxygenations.

By this time, Weiland’s influence was waning (he died in 1957), adding momentum to further initiatives to characterise the relevant biochemistry of biooxygenation. As often happens in scientific research, the pervasive misconception fostered by Weiland’s panjandrum was finally challenged simultaneously by two different scientists working on two completely different enzyme-catalysed reactions, albeit using the same technical approach based on the respective outcomes of ^18^O_2_ and H_2_^18^O incorporation studies. Thus, it was that in 1955 Professor Howard Mason [19] reported that in biotransforming 3,4-dimethylphenol to 4,5-dimethylcatechol, the copper-containing enzyme he called ‘mushroom phenolase’ incorporated one atom of ^18^O_2_, but not the atom of oxygen from H_2_^18^O, into the recovered product (Figure 3C). In order to more correctly reflect the mode of action of the enzyme, Mason subsequently introduced the terms ‘oxygen transferase’ and ‘mixed-function oxidase’ to describe this newly recognised biochemical reaction [20], Within days of Mason’s pioneering studies being published, Professor Osamu Hayaishi [21] used the same experimental technique to confirm that a partially purified non-haem iron-dependent enzyme isolated from an environmental isolate incorporated both atoms of ^18^O_2_ when biotransforming catechol to cis,cis-muconate (Figure 3D). Again, there was no equivalent outcome recorded when the enzyme, which he initially called ‘pyrocatechase’, was tested with H_2_^18^O. As some clarity began to emerge regarding the comparative outcomes of these different molecular oxygen-dependent enzymes, Hayaishi proposed the terms monooxygenase and dioxygenase to describe O_2_-dependent activities that introduce, respectively, one or both atoms of oxygen into relevant products [22]. These pioneering advances by Mason and Hayaishi were quickly followed by other related studies [23,24] that confirmed that the biotransformation of tryptophan to N-formylkynurenine by liver microsomal preparations, an outcome first reported, although not characterised over 20 years previously, was in fact catalysed by tryptophan pyrolase (tryptophan 2,3-dioxygenase (Figure 3E), which unlike Hayaishi’s dioxygenase, proved to be dependent on the tightly-bound prosthetic group iron-protoporphyrin IX (haem iron) to promote the relevant biooxygenation. The spectral characteristics of this strongly-coloured prosthetic group, most notably its characteristic response to exposure to carbon monoxide, led to it being termed P450, which was later changed to cytochrome P450 [25]. The ability to distinguish between the coordinated iron nature of this newly discovered haem iron microsomal enzyme and the previously reported non-haem iron-dependent nature of ‘pyrocatechase’ was based on the absence of any effect of either dialysis or exposure to known chelating agents of unbound iron such as bipyridyl and phenanthroline.

It was against this background of the opening up of oxygen-dependent enzymology as a new intellectually exciting area of biochemistry that Professor Irwin Gunsalus, the undisputed initiator of research into the microbial biodegradation of camphor with its concomitant dependence on the enantiocomplementary DKCMOs, chose to commence his seminal studies at Illinois University in the late 1950s. As head of the Biochemistry Division in the Department of Chemistry, Gunsalus was particularly intrigued by biooxygenation, because although the thermodynamics of the oxidation of most biological molecules by O_2_ are favourable, O_2_ itself is a relatively inert molecule. This results from there being a large kinetic barrier to these reactions. This results from the double-radical nature of paramagnetic ground-state O_2_ which dictates that reactions with biological molecules that are characterised by paired outer orbital electrons is a spin forbidden process. What some might consider the somewhat unusual choice by Gunsalus of camphor as the test substrate with which to explore biooxygenation was again prompted by his astute conceptual grasp of principles. Although a relatively rare compound in nature [26], the fact that the shed leaves from a camphor tree (*Cinnamomum camphora*) growing in a small evergreen plantation on the Urbana-Champaign campus at Illinois University did not result in any detectible level of camphor (‘that little grease-ball’) in the surrounding soil signalled to Gunsalus that there must be an environmental competence to ensure that the C10 bicyclic terpene could gain entry into the global biogeochemical carbon cycle. However, camphor is not a ‘simple’ organic compound for two principal reasons. Firstly, the biterpene is a chiral molecule, most often encountered, including in the camphor tree, as a mixture of two enantiomeric forms. Secondly, its bicyclic nature clearly poses significant biochemical challenges, requiring two carbocyclic ring cleavage reactions, intimating that multiple biooxygenating enzymes may be required, a possibility which in fact only served to increase Gunsalus’s interest in the molecule significantly. Thirdly, with hindsight (always 20/20 vision), camphor may not have been the best choice for a study of biodegradation because of the complex nature of the relevant catabolic pathway which results from a combination of firstly the involvement of isoenzymes dictated by the chiral nature of camphor, secondly the catholic substrate specificity of some of the participating enzymes, and thirdly the complex patterns of transcriptional control that regulate the pathway as a whole (vide infra).

Gunsalus’s programme of camphor-based research was initiated by studies in which the (+)-enantiomer of the bicyclic terpene was deployed as the sole carbon source in a minimal medium inoculated with an environmental isolate sourced from the small heavily polluted stream that ran through Boneyard Creek, located near to his house on the Urbana-Champaign campus of Illinois University. The isolate, originally designated as P, then PpG1, proved to be a bacterium still known to this day as *Pseudomonas putida* ATCC 17453 (NCIMB 10007). Having graduated from a research background of processing kilograms of microbial biomass to isolate µg amounts of lipoic acid [27,28], Gunsalus believed in the value of scale. Consequently, studies commenced to isolate and characterise the metabolites of (+)-camphor that accumulated in 100 litre batches of spent medium recovered after the culture had reached maximum biomass yield on entering into the late log phase of growth, a scale-up operation placed in the capable hands of Rene DuBus. The value of this approach was greatly enhanced by Gunsalus’s enthusiasm for interdisciplinary research, as evidenced in this particular case by collaboration with another academic based in the Department of Chemistry at Illinois University, the distinguished organic chemist ‘E.J.’ Corey (Nobel laureate in Chemistry, 1990). Their joint efforts resulted in a seminal paper published in October 1959 [29] which is notable both for the scope of the catabolic pathway that the characterised metabolites implicated (Figure 2), but also the fact that the identities of all the reported metabolites are still considered correct to this day. Those identified metabolites included 5-*exo*hydroxycamphor, 2,5-diketocamphane, 2-oxo-Δ^3^-4,5,5-trimethylcyclopentenyl acetic acid (OTE), and the δ-lactone of 5-hydroxy-3,4,4-trimethyl-Δ^2^-pimelic acid; collectively, they enabled Gunsalus and Corey to put forward their perceptive proposal for ‘a succinct process for the cleavage of both carbocyclic rings of (+)-camphor’.

Given Corey’s insight that hydrolytic cleavage of both the characterised bicyclic and monocyclic ketone intermediates was not possible on mechanistic grounds, allied to Gunsalus’s newly-acquired enthusiasm for embracing oxygen-dependent biochemistry, attention was then directed to a search for cell-free biooxygenating systems isolated from (+)-camphor-grown *P. putida* ATCC 17453 able to catalyse the relevant lactone-forming reactions [30]. The isolated bicyclic camphor metabolite 2,5-diketocamphane was used as the test bicyclic ketone substrate to probe extracts prepared from cells grown on into late log phase in a (+)-camphor-based minimal medium. This led to the identification of conditions that promoted the production and trapping of monocyclic OTE, itself a transitory intermediate formed from the equivalent initially formed highly labile lactone, characterised as 5-keto-1,2-campholide. Most significantly, OTE production was detected ‘only when the extract was supplemented with NADH in the presence of oxygen’. Gunsalus introduced the term ‘ketolactonase, an enzyme for cyclic lactonization’ to describe the detected activity, which is currently referred to as 2,5-diketocamphane 1,2-monooxygenase (EC 1.14.14.108), a Type II Baeyer–Villiger monooxygenase (BVMO, [31]). This ground-breaking result credits Gunsalus’s ‘ketolactonase’ as being the first characterised BVMO enzyme, because although other equivalent lactonizations were known from the extensive prior studies to characterise steroid biotransformation [17,18], none of the corresponding enzymes had been identified: indeed, at the time the relevant identified steroid metabolites were assumed to have resulted from the action of water-dependent biocatalysts. Gunsalus’s seminal characterisation of the key initial lactonizing step in the pathway for the degradation of (+)-camphor that served to facilitate transformation of the relevant bicyclic intermediate 2,5-diketocamphane into the corresponding monocyclic metabolite OTE [29,30] were subsequently confirmed [32]. A related preliminary study undertaken with *P. putida* ATCC 17453 established that an equivalent enantiocomplementary pathway was induced by growth of the bacterium on (−)-camphor [33], although it was three decades later (vide infra, [34]) before sufficient interest returned to conduct a fuller study of the relevant enzyme 3,6-diketocamphane 1,6-monooxygenase (EC 1.14.14.155).

Attention then moved to isolating the novel ketolactonase from (+)-camphor-grown cells of *P. putida* ATCC 17453 and subsequently exploring functional aspects of its molecular mode of action in a series of relevant papers published in the mid- to late-1960s [35,36,37,38,39,40,41,42,43]. A preliminary study was undertaken aimed at developing a successful ion exchange-based chromatographic separation protocol [36], which was then deployed to isolate the (+)-camphor-induced ketolactonase enzyme [37]. The partially purified enzyme, initially referred to as *E_2_*, was reported to be an 80 kDa monooxygenase based on quantitative amino acid analysis, a prediction that subsequently proved to be accurate (vide infra [34]). Further, the enzyme was deemed to be monomeric because the molar content indicated the presence of only a single N-terminal methionine residue [38], although it was subsequently revised to be homodimeric, but only nearly 30 years later [34]. Significantly, this would prove to be the first of a succession of misconceptions that have dogged not only 2,5-, but also 3,6-DKCMO as their chequered history has progressively unfolded over the last six decades. Because the initially prepared active fractions of the enzyme were noticeably yellow in colour, it was assumed that the responsible enzyme would contain bound flavin, making it as such a true flavoprotein. However, the significance of fact that the yellow flavin moiety readily separated to leave a colourless apoenzyme throughout the later stages of the purification protocol was not acknowledged at the time. No doubt influenced by the recently reported discovery of the involvement of metal ions with various O_2_-dependent enzymes, that initial study of *E_2_* reported that the purified enzyme contained both non-haem and haem iron (iron-protoporphyrrin IX) as well as a bound flavin moiety identified as FMN. The chelating agent bipyridyl was claimed to have a significant inhibitory effect, which was consequently interpreted to indicate the active involvement of the detected non-haem iron in the catalytic activity of *E_2_*. Consequently, the enzyme was deemed to be ‘a flavoprotein containing both tightly-bound FMN and iron in the active site’. Like the proposal that *E_2_* was monomeric, both of these proposals have subsequently proved to be incorrect, and while the proposed involvement of iron was withdrawn within 5 years, the misconception that 2,5-DKCMO is a true flavoprotein had to wait almost 50 years before being finally corrected.

**Figure 4:**
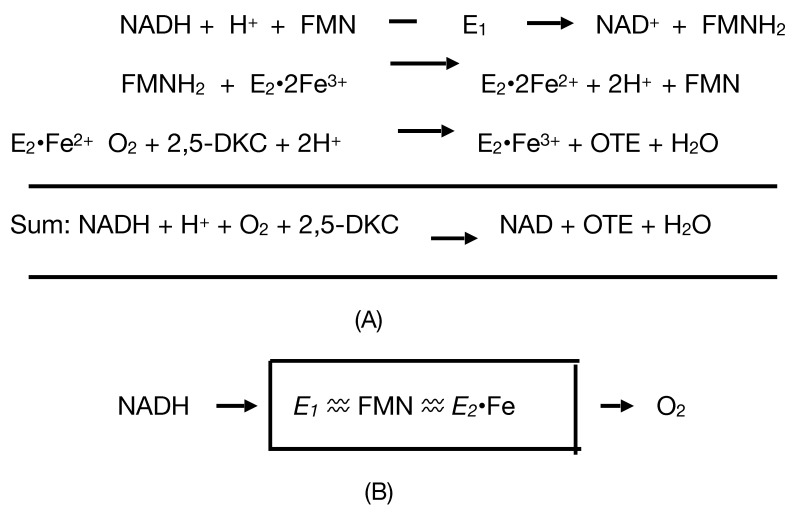
(**A**). Proposed reaction sequence for the combined action of *E_1_* and *E_2_* [41]. (**B**). Cartoon depicting the proposed functional complex between *E*_1_ and *E*_2_ [41]. *E*_1_ = NADH oxidase; *E*_2_ = 2,5-DKCMO; 2,5-DKC = 2,5-diketocamphane; OTE = 2-oxo-△^3,4^-4,5,5-trimethylcyclopentenylacetic acid.

Considerable attention was also focused on another protein that could be isolated and purified from cells harvested during late log phase growth in a (+)-camphor-based minimal medium. Interest in this enzyme, initially referred to as *E_1_*, resulted from the belief that it served the redox partner able to transfer the reducing power necessary for *E_2_* to function as a monooxygenase. Indicative of the fast-moving nature of the research, the enzyme was successively formerly named as FMN reductase [30] and then FMN-coupled NADH oxidase [37] before being finally renamed as NADH dehydrogenase [40]. The changes in name were accompanied by other relevant changes. Rather than the earliest reported value of 50 kDa [37], the enzyme was subsequently claimed to have a mean MW of 36 kDa calculated from sedimentation velocity data, and to be monomeric based on a molar content of a single N-terminal methionine residue by amino acid analysis [38]. However, as occurred with *E_2_*, this form of analysis wrongly concluded that *E_1_* was monomeric and it was nearly half a century later before subsequent research finally established unequivocally that it is a 2 × 18.5 kDa homodimer [44]. In this respect it is significant that the University of Illinois studies of the enzyme, as with *E_2_*, predate the development of SDS-PAGE as a technique to investigate the subunit structure of macromolecules [45]. In common with camphor 5-monooxygenase (cytochrome P450 monooxygenase, EC 1.14.15.1 [46]), the initiating enzyme of the camphor degradation pathway of *P. putida* ATCC 17453, *E*_1_ was shown to contain binding sites for both FMN and FAD. While the incorrect contemporaneous belief that *E_1_* was a monomer resulted in an inaccurate assessment of the actual molar content of both flavin moieties [47], accompanying preliminary kinetic studies [40] did correctly predict that only FMN was actively involved in transferring reducing power from NADH to *E*_2_. Consequently, it was speculated [40] that the bound FAD may serve some sort of support role to enhance the coupling of *E*_1_ and *E*_2_ and hence increase the catalytic efficiency of proton exchange within the loosely bound complex, although no supporting evidence for this proposal was presented either at the time or subsequently. Gunsalus used these various findings to develop a simple model [41] to represent how *E*_1_ and *E*_2_ complement each other functionally as redox partners in an electron transport complex dependent on the involvement of both a bound flavin moiety and non-haem iron to biooxygenate 2,5-DKC to the transitory lactone product 5-keto-1,2-campholide that then spontaneously hydrolyses to yield OTE, another relatively unstable pathway intermediate (Figure 4A). The model proposed that the two participating enzymes are only loosely coupled to form a fragile complex in vivo, with the bound FMN moiety of *E*_2_ acting as a sort of molecular bridge (Figure 4B). Interestingly, haem iron was not assigned any role in the proposed complex despite its presence being reported in *E*_2_ [37]. Some support for these proposals was obtained when a subsequent modified purification procedure developed by the Illinois researchers [41] succeeded in purifying an active camphor lactonizing *E*_1_–*E*_2_ complex. Again, *E_2_* was deemed to be a functioning true flavoprotein containing bound FMN, an errant assumption that remained uncorrected in the literature for almost half a century (vide infra).

Considered in the context of the flurry of interest caused by the newly recognised involvement of various forms of iron in bioxygenation reactions, including the haem iron-dependency of camphor 5-monooxygenase, the initiating enzyme of the camphor degradation pathway [39], and the claimed inhibitory effect of the iron chelating agent bipyridyl on *E*_2_ [37,38], it is perhaps not surprising that Gunsalus initially proposed a role for both haem iron and non-haem iron in purified enzyme preparations of the lactonizing enzyme. However, as typifies the chequered history of the ketolactonases, following the development of an alternative purification protocol which generated a more highly purified preparation of 2,5-DKCMO, allied to the deployment of more powerful analytical techniques, both of these initial proposals were withdrawn soon thereafter by Yu and Gunsalus [43] who reported no detectible trace of iron, and no inhibitory effect of bipyridyl. However, while rebutting the original proposed involvement of iron, this 1969 publication itself introduced its own idiosyncratic canard by reporting the involvement of a third enzyme in the functioning monooxygenase complex, thus expanding it to an *E*_1_ + *E*_2_ + *E*_3_ trimeric assemblage. Again, however, as with the proposed involvement of iron, the requirement for an *E*_3_ protein component to ensure a functional 2,5-DKCMO has not been confirmed subsequently, and it is possible it may simply represent an artefact generated by the particular purification protocol developed by Yu and Gunsalus. Although never actual withdrawn by Gunsalus, significantly there is no referral to the *E*_3_ proposal in relevant reviews published post-1969 [26,48].

**Figure 5:**
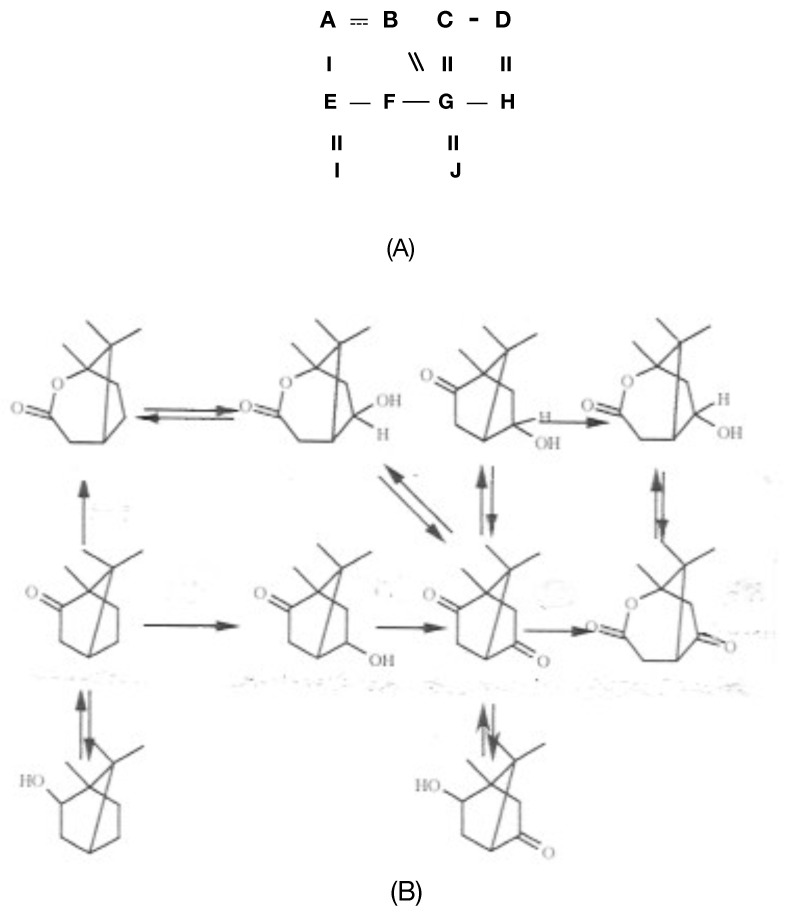
(**A**) The initial cartoon [35], and subsequent proposal (**B**) [38] for a speculative ‘metabolic grid’ for the early metabolism of (+)-camphor based on the characterised reactions catalysed by partially purified preparations of the enzymes camphor 5-monooxygenase, *exo*hydroxycamphor dehydrogenase, *endo*hydroxycamphor dehydrogenase, and 2,5-DKCMO (*E*_2_). A. 1,2-campholide; B. 5-*exo*hydroxy-1,2-campholide; C. 5-*endo*hydroxycamphor; D. 5-*endo*hydroxy-1,2-campholide; E. (+)-camphor; F. 5-exohydroxycamphor; G. 2,5-diketocamphane; H. 5-oxo-1,2-campholide; I. borneol; J. 5-oxoborneol.

Perhaps as much as anything, it was the pace at which the decade of camphor-based research at Illinois took place that inevitably led to a number of errors of interpretation being introduced into the scientific database, albeit in a number of cases these were then subsequently corrected relatively quickly. One further interesting illustration of this sequential timetable of related events resulted from Gunsalus’s initial response to his discovery that a number of the enzymes of the camphor degradation pathway had catholic substrate specificities. Most influential in this respect were studies at Illinois that found that *E*_2_ could catalyse the lactonization not only of 2,5-diketocamphane, but also (+)-camphor, 5-*exo*-, and 5-*endo*hydroxycamphor albeit somewhat less effectively [37]. This outcome, along with the recognition that growth of *P. putida* ATCC 17453 on (+)-camphor also induced reversible 5-*exo*- and 5-*endo*hydroxycamphor dehydrogenases [38], prompted Gunsalus to put forward a highly speculative proposal [38] that the upper part of the camphor degradation pathway may operate as a form of ‘metabolic grid’ (Figure 5). At the time, Gunsalus did not propose any potential advantage(s) that might accrue from *P. putida* ATCC 17453 deploying such an apparently futile reversible interchange of pathway intermediates. As further studies to determine the *K_m_* values of the various implicated substrates for 2,5-DKCMO then established that the 2,5-diketone was likely to be the only viable in vivo substrate, the initial proposal was withdrawn within a matter of months [42]. However, some of the errors introduced into the literature by the frenetic pace of the research at Illinois remained extant in the literature for much longer periods of time. Thus, it was several decades before the significantly over-estimated MW of *E_1_* and the incorrect monomeric status of *E_1_* and *E_2_* were finally corrected (vide infra). However, arguably the most fundamental misconception introduced by the research at Illinois in the 1960s, and one that remained uncorrected for almost half a century, was Gunsalus’s proposal that 2,5-DKCMO (*E_2_*) was a true flavoprotein with bound FMN in the active site. Such was Gunsalus’s reputation and pervading influence as a research scientist that the correct nature of 2,5-DKCMO as an FMNH_2_-dependent two-component monooxygenase that accepts the preformed reduced flavin cosubstrate from a separate distal reductase was only finally confirmed by Iwaki et al.’s seminal study in 2013 ([44]).

With the benefit of hindsight, there were also some significant errors of omission from this body of Gunsalus’s reported research. Probably the biggest lacunae all result directly from the exclusive use by Gunsalus and his fellow researchers of late log phase cultures of camphor-grown *P. putida* ATCC 17453 as sole source of analysed material. While this did serve a typical Gunsalus maxim of optimising the yield of both camphor metabolites and biomass for subsequent analysis and enzyme purification, it left essentially unexplored the significance of any changes that occur in the number, nature, and relative importance of the participating enzymes of the camphor degradation pathway throughout the different phases of batch culture in camphor-based minimal media. This failure of perception is difficult to understand for two principal reasons. Firstly, the phenomenon of growth phase-dependent changes in the specific activity of enzymes had been soundly established in the late 1940s by Professor Roger Stanier [49], a close academic friend of Gunsalus, and secondly and more directly, such changes were actually acknowledged to occur in camphor-grown *P. putida* ATCC 17453 [35,39,42,50]. That Gunsalus failed to appreciate the relevance and implications of those particular changes observed with respect to 2,5-DKCMO, where the monooxygenase (*E*_2_) could be detected throughout trophophasic growth whereas a titre for its redox partner (*E*_1_) could only be detected during idiophasic growth, is particularly puzzling. However, then again, he was not alone, as evidenced by the fact that it was almost half a century after his seminal studies in Illinois before the factors contributing to this mismatch were finally recognised (vide infra).

## 3. The Ketolactonases Revisited but Not Revised—*Déjà vu* in Aberystwyth

Towards the end of the 1960s at Illinois, Gunsalus progressively moved his focus of interest away from the DKCMOs to concentrate almost exclusively on the rapidly expanding field of cytochrome P450-based research, for which camphor 5-monooxygenase, the initiating enzyme of the camphor degradation pathway of *P. putida* ATCC 17453, proved to be an invaluable workhorse [46,51]. As a consequence, after a decade of remarkable achievement, any further progress on characterising the enantiocomplementary DKCMO isoenzymes then effectively stalled, and it was not until almost 20 years later that interest was revived at Aberystwyth University, a renaissance led by Professor Peter Trudgill who had served his apprenticeship as a postdoctoral fellow in Gunsalus’s team at Illinois from 1963 to 1966. As an integral part of a broader platform of research to investigate terpene metabolism by bacteria [26], Trudgill rekindled his interest in the DKCMOs, enzymes that he had extensively researched previously [35,38,40,41], and which consequently he knew remained incompletely characterised. On reflexion, the ensuing years had seen many development that could have contributed positively to this resurgence of the original body of Illinois-based research. Not only had the overarching understanding of microbial enzymology in general increased significantly, but in addition specific methods to isolate, purify and characterise individual enzymes had all progressively advanced [52]. Of particular note, the practice of deploying SDS-PAGE analysis to identify the relevant subunit structure of proteins had been successfully developed [45]. Further, a greater understanding of the dynamic biochemical changes that occur throughout the growth of bacteria in batch culture had emerged [53]. However, the research programme that unfolded at Aberystwyth over the decade 1984–1993 [34,54] was notable for being devoid of a number of these potentially useful concepts and technologies. This may in part have resulted from the pervasive influence of relevant past experience. For instance, reflecting Trudgill’s formative apprenticeship working with Gunsalus at Illinois, the Aberystwyth studies were undertaken exclusively with the recovered biomass from batch cultured *P. putida* ATCC 17453 allowed to progress into the late log-early stationary stage of trophophasic growth in a camphor-based minimal medium. Consequently, the characteristic redox flux of the ketolactonases that results from their now recognised correct status as fd-TCMOs dependent on the rapid diffusional transfer of FMNH_2_ serving as a cosubstrate from a number of different redox partners throughout the growth cycle (vide infra, [8]) was not recognised at the time. Rather, the Aberystwyth studies throughout perpetuated Gunsalus’s historical mantra that each DKCMO is a true flavoprotein with an FMN cofactor tightly bound in the active site which is reduced in situ by a loosely associated enzyme serving as an NADH-dependent oxidoreductase.

Despite these shortcomings, some valuable advances were made in the initial research undertaken at Aberystwyth, resulting jointly from the development of a more refined purification protocol plus the subsequent deployment of SDS-PAGE methodology to characterise the quaternary structure of some, but significantly not all, of the resultant purified proteins [34,54]. Purification of the oxygenating moiety of 2,5-diketocamphane 1,2-monooxygenase (*E_2_*) by a combination of ammonium sulphate fractionation followed by successive ion exchange and affinity chromatographic steps yielded a preparation progressively purified 71.2-fold with a MW of 78 kDa [54]. Trudgill used density gradient ultracentrifugal analysis to predict the MW of the purified enzyme, a result which corresponded very closely with the previous estimated MW of 80 kDa for *E_2_* based on its amino acid content [38]. A significant advance was the recognition, for the first time, that whereas the enzyme was previously predicted to be monomeric [38], SDS-PAGE analysis confirmed that it was a homodimer separable into two apparently identical subunits. Native PAGE separation of a highly purified preparation of the protein, however, yielded two clearly distinct bands, referred to as A and B, both of which had very similar MWs, but which exhibited different isoelectric properties. This unexpected result led Trudgill to postulate that the A and B bands may have resulted from some form of post-translational modification of a single protein type. Rather with hindsight, it is possible to suggest that a more likely explanation for this unexpected native PAGE outcome is that it corresponds to the first indirect evidence for the existence of two different genes on the CAM plasmid coding for isoenzymic forms of the monooxygenase, subsequently identified almost thirty years later (vide infra, [44]) as *camE_25-1_* (*orf* 4) and *cam E_25-2_* (*orf* 22). Another relevant outcome from studying the new highly purified monooxygenase preparation was to confirm Yu and Gunsalus’s 1969 observation [43] that it contained no detectible level of non-haem iron, which finally ended the original considerable speculation [37] for role for the metal ion in the lactonization reaction catalysed by the ketolactonase. While concentrating most effort on *E_2_*, Trudgill’s purification protocols also yielded considerable amounts of an NADH dehydrogenase corresponding to *E_1_* [54].The MW of this protein was estimated to be 36 kDa, but inexplicably no SDS-PAGE analysis was undertaken, and so Trudgill’s misconception introduced 20 years previously [41] that it was a monomeric enzyme was reiterated, only to be finally corrected by Iwaki et al.’s seminal 2013 study [44].

Embracing further advances in protein purification technology, Trudgill then further refined the purification protocol to introduce a Q Sepharose-based FPLC final step [34], the principal advantage of which was to provide access to highly purified homogeneous preparations of both enantiocomplementary ketolactonases. As a consequence, he was able to confirm that 3,6-diketocamphane 1,6-monooxygenase, like the 2,5-ketolactonase isoenzyme, was a homodimer. Again density gradient ultracentrifugal analysis was used to calculate the MW of the purified native enzyme, which was reported to be 76 kDa, designating it as the smaller of the enantiocomplementary proteins. However, the outcome of this was the introduction of another significant contemporaneous error into the database for the enantiocomplementary DKCMOs, because protocols developed almost two decades later in order to recombinantly express the isoenzymes [55,56] clearly demonstrate that 3,6-diketocamphane 1,6-monooxygenase is the larger homodimeric enzyme (2 × 42.3 kDa) compared its enantiomeric partner (2 × 40.5 kDa). That 3,6-DKCMO is indeed larger than 2,5-DKCMO was finally confirmed absolutely in 2013 by comparing the equivalent amino acid sequence data made possible by the subsequent sequencing of the complete CAM plasmid, including the corresponding DKCMO genes by Iwaki et al. [44].

A series of comparative growth studies conducted at Aberystwyth with the separate part-pure commercial grade preparations of the two enantiomers of camphor demonstrated that each antipode could induce significant titres of both enantiocomplementary DKCMOs, which represents the first recorded evidence of the cross-inducibility of the two ketolactonases from camphor-grown *P. putida* ATCC 17453 [34]. The outcome was considered noteworthy because it contrasts sharply with the extremely high enantioselective catalytic activity of each isoenzyme with only the camphor enantiomer from the corresponding chiral series when tested as highly purified homogeneous preparations. This striking contrast, which reflects differing relative selectivities of the respective regulatory and catalytic proteins, has been confirmed by a number of later more comprehensive programmes of relevant research [55,56,57,58,59], albeit the recorded extent of cross-inducibility does vary between the different studies. This latter phenomenon may reflect differences in the relative purity of the various samples of camphor used, which in each case were commodity grade chemicals sourced from different suppliers. Although Trudgill’s cross-inducibility outcomes with the DKCMOs clearly signalled a significant influence of transcriptional regulation on these key enzymes the camphor degradation pathway, the Aberystwyth studies were exclusively conducted with material sourced from biomass harvested in the late log phase of trophophasic growth, thereby failing, as had the prior Illinois studies, to recognise the significant changes that occur in the relative induced levels of these enzymes that occur throughout the growth cycle.

Overall, a pervading sense of *déjà vu* is clearly evident throughout Trudgill’s decade of camphor-based research undertaken at Aberystwyth University. This is most strongly illustrated by the Discussion section of the last relevant paper, published in 1993 [34] which reiterates a number of incorrect proposals directly attributable to Trudgill’s prior active involvement with the studies at Illinois University almost 30 years previously [35,36,37,38,39,40,41,42,43]. Most notable in this respect is the reaffirmation of the proposal first made by Gunsalus [41] that the ketolactonases are true flavoproteins each containing a bound FMN cofactor in the active site. This particular canard subsequently remained unchallenged for another two decades before after almost half a century of mistaken identity, the DKCMOs of camphor-grown *P. putida* ATCC 17453 were finally correctly confirmed in 2013 to be FMNH_2_-dependent TCMOs that function by accepting the pre-reduced flavin as a cosubstrate (vide infra, [44]). However, even as recently as 2015, lingering elements of misconception resulting from Trudgill’s reiterated incorrect claim that the DKCMOs are flavoproteins with bound FMN in the active site remained clearly evident (vide infra, [60]).

## 4. Ketolactonases Reinvented—The Biocatalysis-Led Renaissance

**Figure 6:**
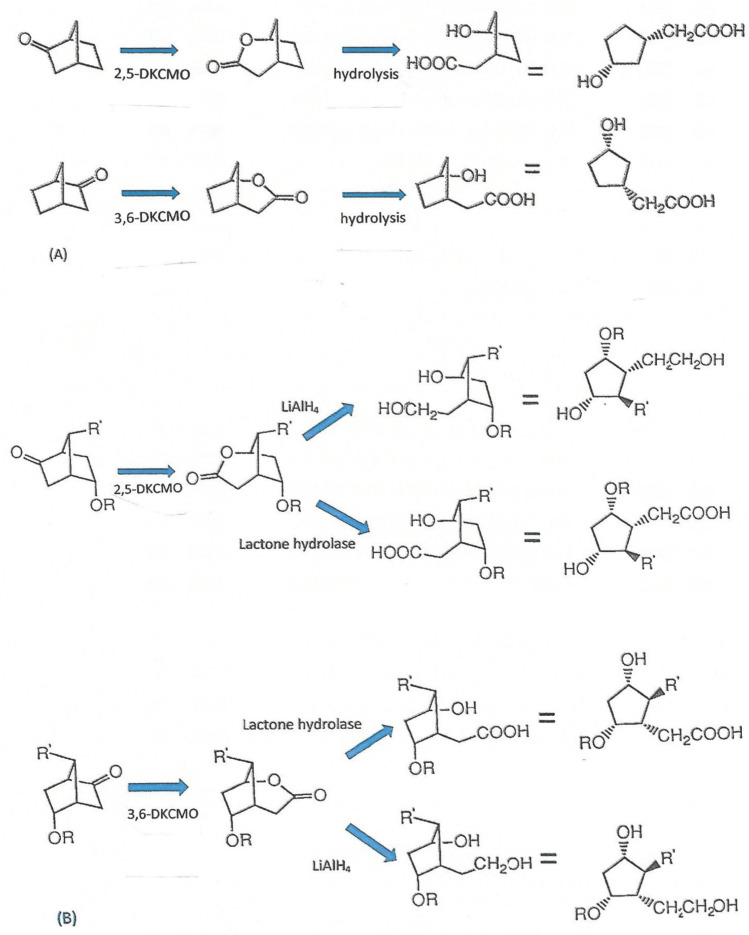
Generation of enantiocomplementary substituted cyclopentanes with multiple chiral centres by deploying DKCMO-catalysed biotransformations of either (**A**) unsubstituted (=2 chiral centres) or (**B**) bisubstituted (=4 chiral centres) bicyclo[2.2.1] ketones as a key step.

Perhaps based on the false premise that most if not all of the fundamental aspects of both the structure and function of the ketolactonases of camphor-grown *P. putida* ATCC 17453 had been resolved, active interest in investigating these aspects of the enzymes ceased following the curtailment of the relevant research programme at Aberystwyth in the early 1990s. Rather, around the same time at Exeter University, a renewed interest in a new facet of the biochemistry of the enzymes grew out of the realisation that the highly specific enantiocomplementary ketolactonases could be successfully deployed as useful biocatalysts (Figure 6) active against a range of both natural and xenobiotic ketone substrates, thereby generating synthons with multiple chiral centres of considerable value for chemoenzymatic synthesis [57,58,61,62,63]. One major outcome of significance from this comprehensive programme of interdisciplinary research headed up by Professor Stanley Roberts was to expand considerably the extant body of knowledge about the idiosyncratic substrate specificities of both the isoenzymes. Recently, this and specific examples of practical outcomes of the programme of collaborative research have been comprehensively reviewed [64].

## 5. New Technologies Herald a New Dawn—The Ketolactonases Reveal Their True Identity

Although evidently successful when deployed using the comparatively small-scale protocols developed at the University of Exeter, it was obvious that the potential to scale up the technology for larger industrial-scale applications would be significantly hindered by reliance on the then established purification protocols, which were both protracted and FPLC-based, and as a consequence, low-yielding [34,62]. This realisation then promoted a new initiative led by Professor Uwe Bornscheuer at Greifswald University to develop suitable vectors to exploit host recombinant expression of the relevant ketolactonase genes as a potential way to generate larger amounts of each biocatalyst. As the ketolactonases were still considered to be true flavoproteins at the time, a successful outcome from the initiative would require both the identification of a suitable complementary NADH-dependent redox partner and the development of an effective coupled-enzyme operating system. Initial studies [55] were focussed on 2,5-diketocamphane 1,2-monooxygenase, for which it was possible to isolate a relevant gene from DNA sourced from the CAM plasmid of *P. putida* ATCC 17453 with the aid of primers derived from GenBank entry AY450285.1 (2003), The gene, now known to correspond to *camE_25-1_*, was then used to generate relatively large amounts of the corresponding protein by exploiting the well-developed *E. coli* BL21(DE3) recombinant expression system. Contrary to expectations, although only the gene coding for the oxygenating moiety of 2,5-DKCMO was expressed in the heterologous host, significant lactonizing activity was recorded with partially purified preparations of the recovered recombinant enzyme, which was established to be flavin-free. Attention then turned to the enantiocomplementary 3,6-DKCMO [56], an initiative facilitated by deploying gene walking PCR from *camE_25-1_* to successfully identify *camE_36_*, the corresponding gene on the CAM plasmid that codes for 3,6-diketocamphane 1,6-monooxygenase (vide infra). Subsequent heterologous expression of *camE_36_* the in the same host again resulted in the unexpected outcome of significant lactonizing activity being recorded with partially purified flavin-free preparations of the recovered recombinant enzyme. These data were interpreted by Bornscheuer as indicating that one or more native activities of the *E. coli* host was serving to complement the cloned ketolactonases by generating FMNH_2_, thereby resulting in the recorded lactonizing activity of both the recombinantly expressed enzymes.

It was these purely serendipitous outcomes of the recombinant expression studies at Greifswald University that prompted the initial realisation that almost half a century of dogma, built on foundations initially promulgated by Irwin Gunsalus at Illinois University, and subsequently reiterated by Peter Trudgill at Aberystwyth University, could actually be wrong. Thus, the Greifswald University data suggested that the ketolactonases of camphor-grown *P. putida* ATCC 17453, rather than being true flavoproteins, may instead be fd-TCMOs, as such corresponding to a few other recently reported bacterial enzymes acknowledged as being a completely novel class of monooxygenases [2]. Undoubtedly the best characterised bacterial fd-TCMOs at that time were the luciferases from *Vibrio harveyi*, *V. fischeri*, *Photobacterium phosphoreum*, *P. legionathi*, and *Xenorhahdus luminescens* [65].The fact that luciferases, like the ketolactonases, are enzymes that function as Baeyer–Villiger monooxygenases was considered by Bornscheuer as indirect support for his radical proposal, as was the fact that bacterial luciferases and the ketolactonases had been grouped together over a decade previously, as so-called Type II BVMOs, on the basis of a number of other shared structural and functional properties [31]. In an attempt to further investigate and characterise the *E. coli*-triggered lactonizing activities of the overexpressed ketolactonases from *P. putida* ATCC 17453, a dual initiative was then launched [66,67] to identify both the nature of the competent FMNH_2_-generating enzyme(s) in *E. coli*, and any equivalent enzyme(s) in *P. putida* ATCC 17453 that would correspond to *E_1_* (vide supra), Trudgill’s apocryphal monomeric 36 kDa dehydrogenase [34,40,41,54], ironically an activity that was transiently referred to over 50 years previously as ‘FMN reductase’ [30]. The functional native *E. coli* activity was successfully identified as the known monomeric 26.2 kDa FMN-dependent enzyme Fre, which can serve a dual purpose ferric-flavin reductase role [68]. However, while a number of putative flavin reductase genes were identified in the genome of *P. putida* ATCC 17453 by CODEHOP PCR [69], including a 28.5 kDa enzyme with 83% sequence homology to the ferric-flavin reductase FprB from *P. putida* KT2440, ultimately the latter programme of research was unsuccessful because none of the identified candidate reductase genes yielded any equivalent titres of active protein when deployed in the *E. coli* BL21(DE3) recombinant expression system [66]. Consequently, further interest in the programme subsequently declined at Greifswald University, and irrefutable confirmation both of the status of the ketolactonases of *P. putida* ATCC 17453 as fd-TCMOs, and identification and characterisation of the relevant native flavin reductases then had to await subsequent inputs, initially by Iwaki et al. [44], and then more comprehensively by Willetts and Kelly [47,70].

The definitive evidence that the ketolactonases from camphor-grown *P. putida* ATCC 17453 are indeed FMNH_2_-dependent Class C TCMOs was provided by a joint initiative led by Professors Hiroaki Iwaki and Yoshie Hasegawa (Kansai University), and Peter Lau (McGill University). Their seminal paper (Iwaki et al. [44]) published in May 2013 describes the isolation and full characterisation of a 37 kDa FMNH_2_-donating enzyme present in cells of the bacterium harvested in the late log-early stationary phase of growth on (+)-camphor, and confirmed that it could facilitate efficient lactonization reactions catalysed by purified preparations of both ketolactonases. These characteristics of the enzyme, which was given the trivial name Fred (Flavin reductase), make it a strong candidate both for the activity in *P. putida* ATCC 17453 serendipitously predicted by Bornscheuer [54,55], and for the enzyme that had been misidentified throughout both Gunsalus’s and Trudgill’s prior studies (vide supra) as being a monomeric 36 kDa protein variously referred to as FMN reductase [30], *E_1_* [36], NADH oxidase [37,54], and NADH dehydrogenase [34,40,41]. Purified samples of Fred prepared from late log-early stationary phase cells grown on (+)-camphor were sequenced, and that data then used to design probes that confirmed that the corresponding gene was located on the chromosomal DNA rather than the CAM plasmid. Analysis of the gene predicted a corresponding protein product of 170 amino acids, including the presence of the characteristic flavin reductase motifs GDH [71] and YGG [72]. The predicted *M_r_* of the protein was 18,466. Unlike the equivalent chromosomal gene coding for a 28.5 kDa reductase identified by the earlier research undertaken at the Greifswald University [66], subsequent cloning and over expression of the gene coding for Fred generated large quantities of fully functional protein, enabling the enzyme to be highly purified and then extensively characterised. These studies established that the active form of the enzyme was an NADH-specific 2 × 18.5 kDa homodimer. The data from relevant kinetic analysis of purified samples of enzyme confirmed that while FMN, FAD and Rf can all serve as substrates, FMN was the flavin most efficiently reduced by Fred. Collectively, these outcomes confirm that Fred corresponds to a Class II non-flavoprotein reductase [3], which operate a sequential rather than a ping-pong reaction mechanism [2,4] to transfer generated FMNH_2_ to serve as a cosubstrate for redox partner enzymes by rapid free diffusion. Comparison with protein databases confirmed that Fred appears to most closely resemble NTA-MoB, an established flavin reductase partner of nitrilotriacetate monooxygenase from *Chelatobacter heintzii* ATCC 29,600 [73] which was one of the first FMNH_2_-dependent TCMOs to be identified in the mid-1990s.

**Figure 7:**
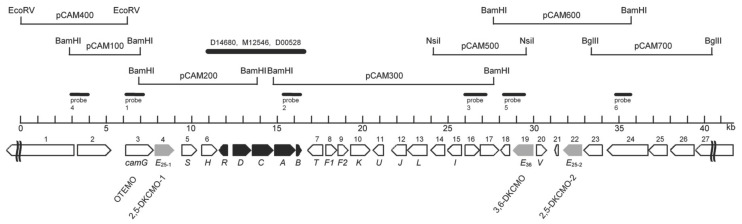
Localization of additional genes and predicted open reading frames (ORFs) flanking the established initial genes of the camphor *camDCAB* operon and its repressor, *camR*, on an~40.5-kb established sequenced region of the CAM plasmid of *P. putida* ATCC 17453. The predicted ORFs or genes are sequenced numbered from 1 to 27, except for the established *camRDCAB* genes, which are shaded in black. The numbered from 1 to 27, except for the established *camRDCAB* genes, which are shaded in black. The orientation of the arrows indicates the direction of gene transcription. The candidate genes of this study (*camE*_25–1_, *camE*_25–2_, and *camE*_36_) representing the three diketocamphane monooxygenase (DKCMO) isozymes are highlighted in grey. The previously established OTEMO-encoding gene been designated *camG* in accordance with the respective catabolic step. Figure reproduced from Iwaki et al. [44] with permission. American Society for Microbiology, licence number 4487631509592.

As well as their seminal contribution by confirming for the first time the true nature of the ketolactonases of *P. putida* ATCC 17453 as fd-TCMOs, the outcomes from Iwaki et al.’s research [44] also significantly increased knowledge both about the CAM plasmid as a whole, and the specific concatenated *cam* operon region which codes for all the enzymes of the camphor degradation pathway. One novel notable outcome was that contrary to earlier predictions [74,75], the plasmid proved to be a large linear 533 kb double-stranded transmissible genetic element, thereby updating its previously perceived circular nature. Their use of a combination of standard cloning and sequencing techniques to search for individual genes also proved rewarding (Figure 7). A corresponding gene for the oxygenating moiety of 3,6-diketocamphane 1,6-monooxygenase (*camE_36_* [*orf19*]) was located within a contiguous 40.5 kb region of the established *camRDCAB* locus of the CAM plasmid, confirming an earlier relevant study at the Greifswald University [56]. However, by way of contrast, the equivalent search that focused on 2,5-diketocamphane 1,2-monooxygenase yielded an unexpected novel outcome by identifying two separately located corresponding genes which were designated *camE_25-1_* (*orf4*, coding for 2,5-DKCMO-1), and *camE_25-2_* (*orf22*, coding for 2,5-DKCMO-2). Thus, for the first time, it became apparent that 2,5-DKCMO, which had historically been considered to be a single protein entity, actually comprised two distinct isoenzymic forms of the same enzyme. This novel outcome then raised the possibility that the detection in 1986 by Taylor and Trudgill [54] of two electrophoretically separable forms of this enzyme (A and B), could have represented the first, albeit unrecognised, evidence of the isoenzymic status of 2,5-DKCMO, rather than corresponding to two different post-translationally modified forms of a single protein as proposed at the time. As such it represents another example of how historical misinterpretation of data has dogged the ketolactonases for several decades.

**Figure 8:**
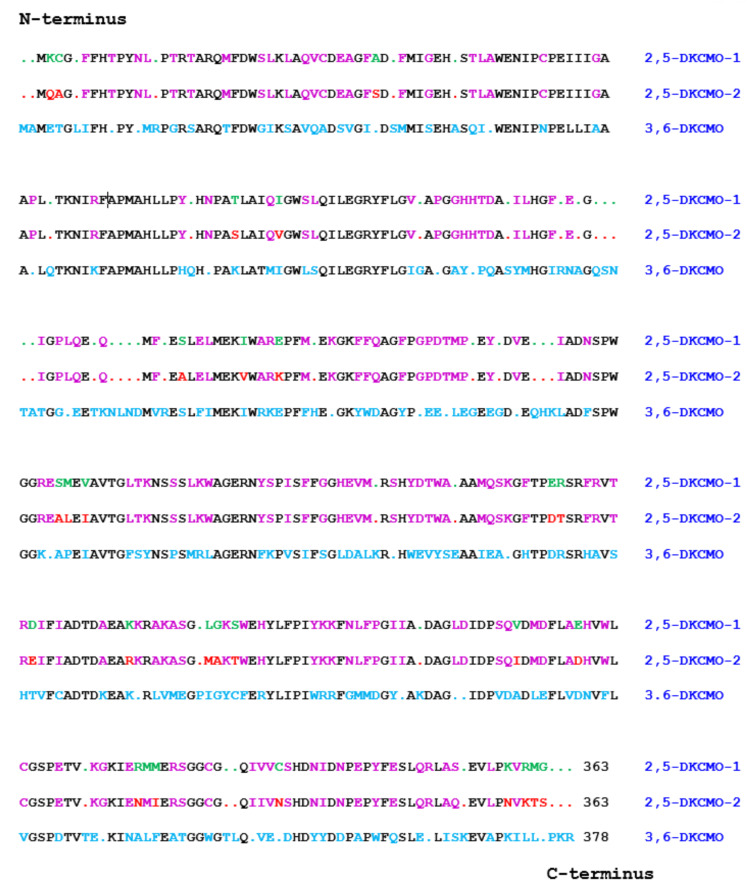
Protein sequence alignment of the oxygenating subunits of 2,5-DKCMO-1 (*orf-4*, *camE_25-_*,) 2,5-DKCMO-2 (*orf-22*, *camE_25-2_*) and 3,6-DKCMO (*orf-19*, *camE_36_*). Amino acid residues: common to 2,5-DKCMO-1, 2,5-DKCMO-2, and 3,6-DKCMO = **black**; common to 2,5-DKCMO-1 and 2,5-DKCM-2 = **magenta**; exclusive to 2,5-DKCMO-1 = **green**; exclusive to 2,5-DKCMO-2 = **red**; exclusive to 3,6-DKCMO = **blue**.

A number of direct and indirect consequences subsequently resulted from Iwaki et al.’s seminal 2013 studies. An obvious direct consequence was that the identification and sequencing of the *camE_25-1_*, *camE_25-2_*, and *camE_36_* genes presented the first opportunity to compare the complete primary structure of the ketolactonases induced in camphor-grown *P. putida* ATCC 17,453. Translation of the relevant nucleotide sequences confirmed that 3,6-diketocamphane 1,6-monooxygenase is the larger protein. It comprises 378 amino acid residues compared to the 363 amino acid residues that characterise the two proteins coded for by the *camE_25-1_* and *camE_25-2_* genes. This outcome served to correct a further example of the misinterpretations of data that are prevalent throughout the history of characterising camphor metabolism by *P. putida* ATCC 17453, namely the proposal made 20 years previously by Jones et al. [34] that 3,6-DKCMO is the smaller protein. Rather than using direct corresponding gene sequence data, Jones et al. had reached their earlier conclusion indirectly by comparing the sedimentation characteristics of purified preparations of both ketolactonases when subjected to density gradient ultracentrifugation, which clearly had yielded a less reliable outcome. Further in-depth analysis of the sequence data (Figure 8) using standard alignment tools has served to emphasise the considerable similarity between all the three sequences. While the two isoenzymic 2,5-diketocamphane 1,2-mono-oxygenases exhibit the highest alignment score (1852 using BLOSUM62), the equivalent scores for both isoenzymes when compared to 3,6-diketocamphane 1,6-monooxygenase (961 and 965) are very similar. The closely related nucleotide sequences of the *camE_25-1_*, *camE_25-2_*, and *camE_36_* genes suggests that they probably arose by gene duplication and subsequent divergence [76]. That being the case, an interesting future study would be to compare the outcomes of the resolved natural evolutionary relationships between 2,5-DKCMO-1, 2,5-DKCMO-2, and 3,6-DKCMO, and a multi-round programme of directed evolution of the corresponding genes [77].

**Figure 9:**
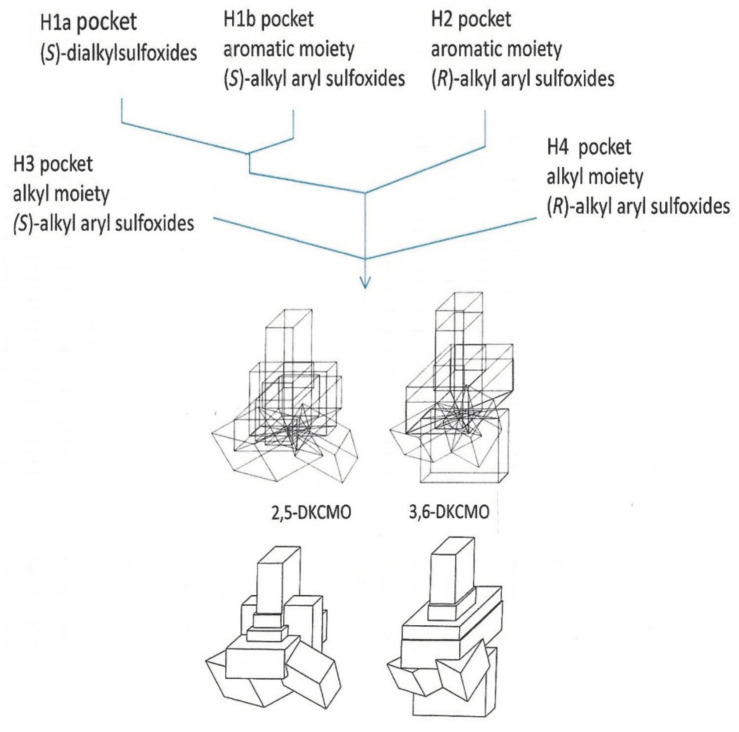
Rationale for superimposing the minimalised structures of the sulfoxides formed by 2,5-DKCMO and 3,6-DKCMO, and the resultant total dimensions (‘cubic space’) of the relevant enzyme active sites.

An indirect consequence of Iwaki et al.’s pioneering 2013 studies [44] was that the resultant knowledge of the primary structures of all the DKCMO isoenzymes resparked interest in determining their corresponding three-dimensional structures. Historically, surprisingly little relevant information on structural features of the isoenzymic ketolactonases had been published. The one exception was a 1998 study [59] that deployed so-called ‘cubic space models’ [78] to develop three-dimensional (3D) representations of the active sites of the enantiocomplementary DKCMOs based on the outcomes of biotransformation conducted with twenty-three organosulphide substrates (Figure 9). This study had its strengths and weaknesses. With hindsight, it has to be appreciated that the representation generated for 2,5-DKCMO represent a collage of the outcomes from 2,5-DKCMO-1 and 2,5-DKCMO-2. However, the study has significant merit because the DKCMOs, like other enzymes, undergo sequential structural changes during catalysis [79], and a strength of this approach is that the resulting models reflect the relative extent of relevant dynamic structural changes for the DKCMOs. It was also clear from this 1998 study that the substrate-binding active site of 3,6-DKCMO was significantly larger than that of 2,5-DKCMO, which may correspond with 3,6-DKCMO being confirmed to be the larger protein by Iwaki et al.’s study [44]. 

Iwaki et al.’s intimate knowledge of the CAM plasmid and relevant recombinant expression protocols able to deliver the large amounts of DKCMO protein that would be necessary to generate diffraction-grade crystals resulted in them being recruited into a collaborative X-ray crystallographic study with Professor Jennifer Littlechild and Dr Michail Isupov Exeter University) to determine the 3D structure of 3,6-diketocamphane 1,6-monooxygenase, an undertaking that ultimately proved to be controversial [60]. The benchmark for such studies of the O_2_-dependent enzymes of camphor-grown *P. putida* ATCC17453 had been set a number of years earlier by Thomas Poulos’s high-level resolution of the crystal structure of camphor 5-monooxygenase [80]. Capitalising on experience gained from a series of unsuccessful attempts previously undertaken with 3,6-DKCMO [81,82,83], Isupov et al. used the known structure of the α-subunit of the luciferase from *Vibrio harveyi* in combination with molecular replacement and MAD phasing techniques and relevant density modification to generate a crystal structure of the ketolactonase (PDB code 4UWM [60]). Although the choice of the α-subunit of the luciferase from *Vibrio harveyi* to resolve the phase problem represented the best available sequence homology (16%) that could be sourced at the time [84], this is well within the so-called ‘twilight zone of protein sequence alignments’ which make comparative structure predictions unreliable [85]. Further, it was known that the two proteins exhibit some significant structural and functional differences [86,87], which may in part explain why Isupov et al. reported that their attempts to model active site binding of a flavin moiety proved to be problematical. Additionally relevant in this respect is that the modelling studies were undertaken with FMN, a flavin for which functional binding is only relevant for true flavoproteins where FMN serves as a tightly-bound cofactor. For 3,6-DKCMO, convincingly shown to be an FNMH_2_-dependent TCMO in almost two years previously by Iwaki et al. [44], the relevant flavin moiety is performed FMNH_2._ which functions as a fully reduced redox flux cosubstrate, generated by one or more competent partner flavin reductases (vide infra). Despite Iwaki, Hasegawa and Lau being cited as coauthors, this misconception regarding the correct nature of the relevant flavin moiety for 3,6-DKCMO is reflected in Isupov et al. [60] by the multiple inclusion of the term ‘cofactor’ which contrasts with a total absence of the term ‘cosubstrate’. Additionally relevant in this context are both the well characterised difference in three-dimensional shape between FMNH_2_ and FMN [87,88], and the fact that FMN exhibits random binding to fd-TCMOs [2], as evidenced specifically for 3,6-DKCMO by a kinetic study which confirmed a 500-fold difference in the binding capacities of FMNH_2_ and FMN [47]. Consequently, another flawed chapter has been added to the history book for the ketolactonases, and a revision must await the outcome of a future study based a combination of factors. Firstly, the use of a closer protein match than the α-subunit of luciferase to resolve the phase problem, secondly a correct understanding of the role of FMNH_2_ in the generation and deployment of ‘active oxygen’ by the DKCMOs, and thirdly an appreciation of the functional domain movements that characterise the mode of action of BVMOs [89].

There was one final bizarre twist to this multi-national collaborative study of 3,6-DKCMO that is directly related to the extensive knowledge Iwaki, Hasegawa and Lau had gained from their prior investigation [44] of camphor-grown *P. putida* ATCC 17453. Significantly, their 2013 study reported the complete nucleotide sequence of a concatenated 40.5 kb operon on the CAM plasmid that coded for all the degradation pathway enzymes necessary to catabolise the C10 terpene via OTE and Δ^2-5^-3,4,4-trimethylpimelyl-CoA to the key central intermediary metabolites acetyl-CoA and isobutyl-CoA (Figure 2). However, another important outcome of their 2013 study was that the sole redox partner identified as providing the requisite FMNH_2_ cosubstrate for the DKCMO isoenzymes to function as fd-TCMOs was the chromosome-coded flavin reductase Fred. This exception is important because, taken at face value, Iwaki et al.’s results imply that the CAM plasmid of *P. putida* ATCC 17453 cannot function as a fully autonomous metabolic entity (vide infra), making it a notable exception to other then known bacterial catabolic plasmids [8,75,90]. This perceived deficiency then promoted Littlechild and Isupov, the two cited lead authors of the 2015 study of CAM plasmid-coded 3,6-DKCMO [60], to include an unsupported claim to have ‘now identified a flavin reductase adjacent to the 3,6-DKCMO gene on the CAM plasmid’. However, the claim by Littlechild and Isupov was clearly fallacious given that the complete sequence of the 40.5 kb concatenated region of the CAM plasmid, including the gene coding for 3,6-DKCMO (*orf19*) plus all 18 upstream and 7 downstream proximal *orfs*, had been published 30 months previously by Iwaki, Hasegawa and Lau [44], all of whom are inexplicably cited coauthors of the 2015 study. Fortunately this was one corruption of the DKCMO database that was quickly investigated by the publishing journal, and subsequently corrected by a relevant corrigendum that unreservedly withdrew the unsupported claim and replaced it with the relevant cognisant information [60].

**Figure 10:**
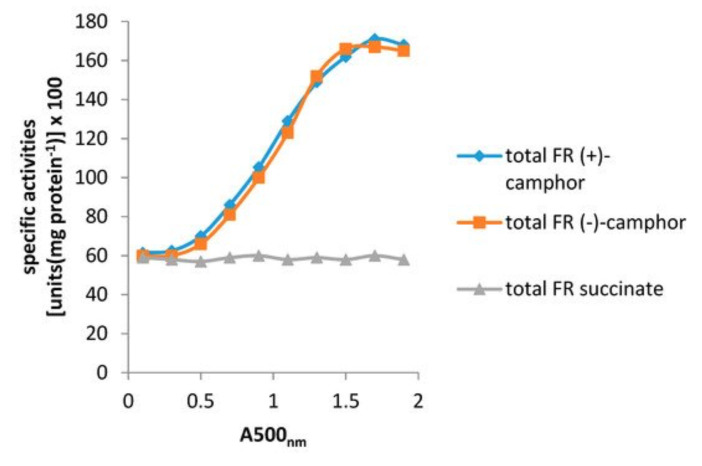
Specific activity of total flavin reductase (FR) throughout the growth of *P. putida* ATCC 17543 on either (+)-camphor, (−)-camphor, or succinate as sole carbon source.

Resolving the issue of the self-sufficiency of the CAM plasmid did, however, trigger a broader based research initiative to investigate factors that control the supply of the FMNH_2_ cosubstrate necessary for the DKCMOs to function effectively as fd-TCMOs. The initial objective of the study was to identify which, if any, indigenous reductases of *P. putida* ATCC 17453 other than Fred can support the ketolactonases [70]. Studies of total flavin reductase activity throughout the growth of *P. putida* ATCC 17453 on either succinate- or (+)- or (−)-camphor-based minimal media confirmed that very similar titres of activity were detectible in the earliest lag phase cells sampled after inoculation into all three media. However, whereas that level remained remarkably consistent in succinate-grown cells throughout trophophasic growth, the flavin reductase titre of cells grown on either (+)- or (−)-camphor-based minimal medium was progressively induced to a maximum 2.8-fold higher level during late trophophasic growth before plateauing after entry into the stationary (idiophasic) phase of growth (Figure 10). This data implied that growth of *P. putida* ATCC 17453 on either enantiomer of camphor generated an equivalent enhanced intracellular demand for FMNH_2_. A combination of gel-filtration chromatography, electrophoresis, and comparative sequence data analysis of purified recovered samples confirmed that two monomeric reductases (Frp1, 27.5 kDa and Frp2, 28.5 kDa) which correspond with the previously characterised chromosome-coded ferric-flavin reductases FprA and FprB from *P. putida* KT2440 [91], were the only relevant activities recorded after growth on succinate. Based solely on relevant MW data, it is possible that Frp2 corresponds to the same previously uncharacterised flavin reductase-type gene detected in the course of the prior CODEHOP PCR studies carried out at the University of Greifswald [66]. Equivalent analysis of the camphor-grown cells confirmed that very similar constitutive titres of Frp1 and Frp2 were present after growth on either camphor enantiomer.

Attention then focused on what other activities were responsible for the significantly up-regulated total camphor-induced reductase activity. This search then identified two principal additional camphor-induced reductase activities, both induced by growth on either terpene enantiomer. Perhaps not unsurprisingly, one of these was confirmed to be Fred, the 37 kDa homodimer reported previously as the sole reductase activity of *P. putid*a ATCC 17453 [44]. More unexpectedly, however, was that the purified monomeric protein responsible for the second additional camphor-induced flavin reductase activity had an N-terminal amino acid sequence consistent with that of putidaredoxin reductase (PdR), a 48.5 kDa protein coded for by the *camA* gene of the CAM plasmid, and known to serve in the role of an FAD reductase as one of the functioning components of camphor 5- monooxygenase [92], but which had not been reported previously to serve as an FMN reductase. Interestingly, the initiation codon GTG for *camA* is a rare start codon, and is thought to be important in the control of PdR abundance. Purified samples of Fred, Fpr1, Fpr2, and most significantly PdR, were then confirmed to be equally active in supporting lactonization reactions with a high degree of enantioselectivity by purified preparations of both DKCMOs. This previously unrecognised ability of PdR to serve a functional role in the redox flux that supports the DKCMOs has significant implications for the metabolic autonomy of the catabolic CAM plasmid of *P. putida* ATCC 17453 (vide infra). That four different identified reductases present in camphor-grown *P. putida* ATCC 17453 can serve effectively as distal sources of the requisite FMNH_2_ for both of the DKCMOs confirms the non-specific nature these redox relationships, as is a characteristic feature of fd-TCMOs [2]. This catholic relationship has been emphasised further by the recent demonstration that biomimetic nicotinamide analogues serving as hydride donors can participate in reductase-free FMNH_2_ generating systems able to support the biooxygenating activity of highly purified 2,5-DKCMO [93].

**Figure 11:**
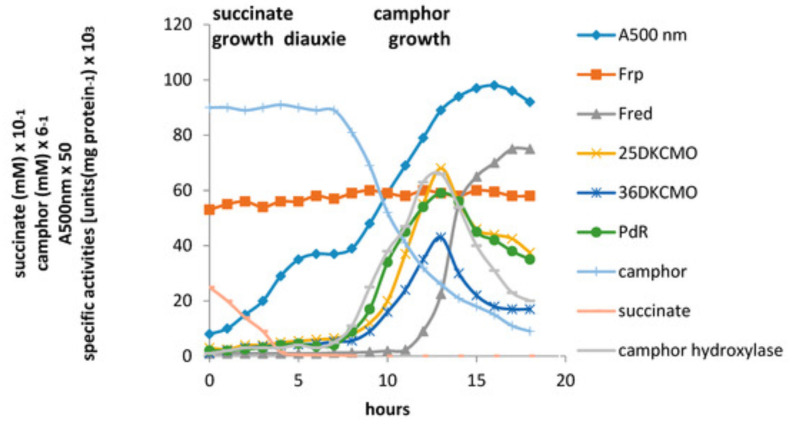
Changes in the optical density (A_500_nm), succinate (mM), (*rac*)-camphor (mM), and the specific activity of key enzymes of (*rac*)-camphor degradation during diauxic growth of by *P. putida* ATCC 17453 on succinate plus (*rac*)-camphor based defined medium. Camphor hydroxylase = camphor 5-monooxygenase.

The relative roles of the each of the identified flavin reductase activities in supporting lactonizing activity by the DKCMOs were then examined in more detail in a subsequent study [47], which both logged the titres of all relevant reductase and oxygenating activities promoted by (*rac*)-camphor as a growth substrate, and additionally characterised relevant kinetic data for the generation of FMNH_2_ and its subsequent transfer to the oxygenating subunits of the DKCMOs. The camphor-promoted events were monitored (Figure 11) by following up on the early observation by Gunsalus et al. [42] that during the diauxic growth of *P. putida* ATCC 17453 on a defined succinate plus (*rac*)-camphor medium, the enzymes of the camphor degradation pathway were only expressed after complete exhaustion of the succinate. With respect to the oxygenating activities, both the timing and extent of induction of 2,5-diketocamphane 1,2-monooxygenase and camphor 5-monooxygenase were very similar in response to the swop to (*rac*)-camphor as the growth substrate. While the enantiocomplementary 3,6-diketocamphane 1,6-monooxygenase followed a very similar time course, the level of induction was consistently lower, confirming some earlier preliminary observations [57,62,63]. Not unexpectedly, the profile of induction of PdR activity coincided exactly with that of the composite camphor 5-monooxygenase, of which the monomeric 48.5 kDa protein is a confirmed functional subunit [94]. Kinetic data derived from characteristic changes in the relevant absorption spectra confirmed conclusively that the highly purified PdR preparation accepted reducing power exclusively from NADH thereby generating bound FADH_2_, which in turn was able to pass reducing power to unbound FMN added subsequently as a cosubstrate. This sequential transfer of reducing power from NADH via FADH_2_ to FMNH_2_ defines a de novo role for PdR, and explains the confirmed ability of a highly purified preparation of the enzyme to function as the effective redox partner for highly purified preparations of both enantioselective ketolactonases [47]. In turn, this is key evidence that the catabolic CAM plasmid of *P. putida* ATCC 17453 can serve as an autonomous metabolic entity (vide infra).

Detailed investigation of Fred, the 37 kDa homodimeric flavin reductase which Iwaki et al. [44] claimed corresponded to the activity variously referred to as *E_1_*, FMN reductase, FMN-coupled NADH oxidase, or NAD dehydrogenase in early studies at Illinois [30,34,36,37,40,41,54], confirmed that it is, like PdR, a camphor-induced enzyme. Initial Michaelis Menton kinetic studies using a highly purified preparation of the enzyme confirmed Iwaki et al.’s original conclusion that Fred can only effectively transfer reducing power from NADH to FMN and shows minimal activity in any combination with either NADPH or FAD or riboflavin. Double reciprocal kinetic plots were then deployed to provide a more detailed understanding of the enzyme, thus confirming that the enzyme is a EC 1.5.1.x-type Class II non-flavoprotein reductase [3,95] that generates FMNH_2_ by a sequential reaction mechanism [2,4].

The titres of Frp1 and Frp2 were remarkably consistent throughout each stage of the progression from succinate-dependent to camphor-dependent growth, confirming the outcome of the earlier growth study [47] which identified that both Frp1 and Frp2 are constitutively expressed activities in *P. putida* ATCC 17453. Kinetic studies confirmed that a purified preparation of Frp1, while totally inactive with NADPH, was able to serve as an effective means of generating FMNH_2_ from NADH while showing minimal and no corresponding activity with either FAD or riboflavin. While Frp2 gave similar outcomes, there was some limited generation of FMNH_2_ from NADPH compared to NADH as an alternative source of reducing power. However, the relative kinetic data suggest that the recorded low level of activity with NADPH is unlikely to be physiologically significant. Double reciprocal plots of relevant kinetic data confirmed that both Frp1 and Frp2, like Fred, are EC 1.5.1.x-type Class II non-flavoprotein reductases [3,95], that generate FMNH_2_ by a sequential reaction mechanism [2,4].

**Figure 12:**
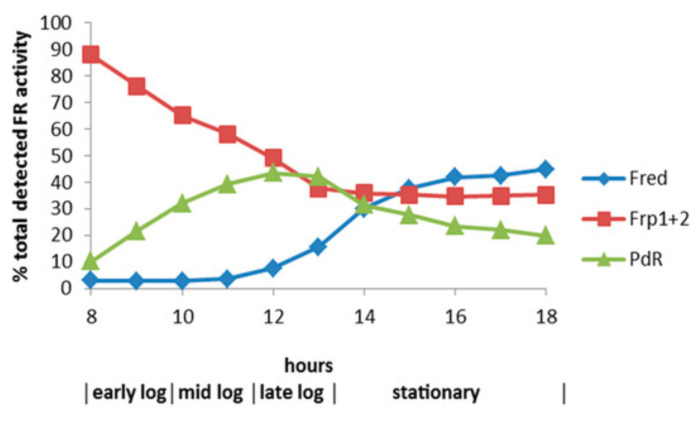
Relative contribution of the different assayed FNR-generating enzymes to the total flavin reductase activity titre throughout the various phases of (*rac*)-camphor-dependent growth of *P. putida* reductase activity titre throughout the various phases of (*rac*)-camphor-dependent growth of *P. putida*.

By reformatting the data from the diauxic growth study, a picture could be gained of the relative contribution of the different assayed FMNH_2_-generating enzymes to the total flavin reductase activity titre throughout the progressive phases of the camphor-dependent growth of *P. putida* ATCC 17453 (Figure 12). The outcome confirmed the initially predominant importance of the combined constitutive Frp1 and Frp2 activities at the onset of camphor-dependent growth. However, this predominance was progressively diminished throughout early and mid log trophophasic growth by the steady incremental induction of the CAM plasmid-coded PdR activity. Thereafter, these three flavin reductase activities progressed to assume approximately equal importance during the late log phase in the growth cycle, before the relative importance of PdR declined significantly as induction of the protein is down-regulated after exit from trophophase into idiophase (stationary phase). Contrastingly, the titre of chromosome-coded camphor-induced Fred only began to assume any importance during the very late log phase of trophophasic growth, but then progressed to become the predominant FMNH_2_-generating activity following entry into idiophase, a profile matched concomitantly by the declining titres of PdR and, as evident from Figure 11, both DKCMOs. This time course for the up-regulation of Fred is more consistent with that of an enzyme involved in secondary (idiophasic) rather than primary (trophophasic) metabolism [95]. It is relevant in this respect that species of *P. putida* are known to produce a wide range of indigenous secondary metabolites [96], including various polyketides [97], for which fd-TCMOs are known to play important roles as ‘tailoring enzymes’ [98,99,100].

**Figure 13:**
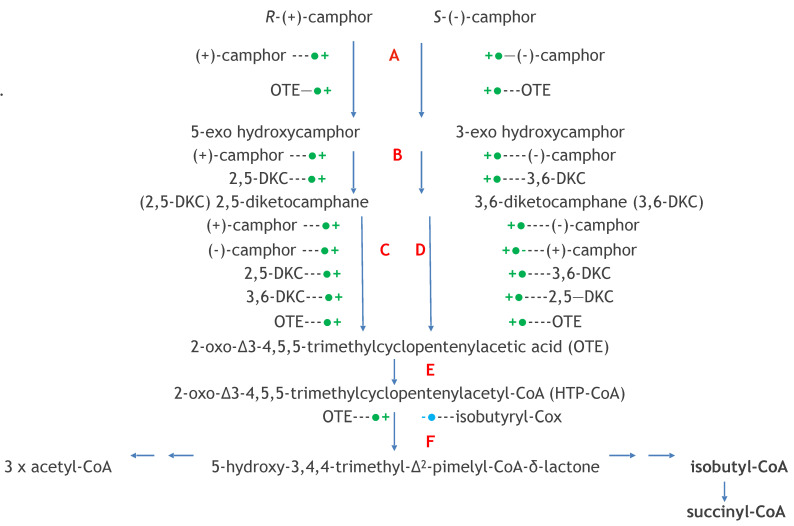
Transcriptional controls of the pathway of (+)- and (−)-camphor degradation in *P. putida* ATCC 17453 ●+ = induction: −● = repression: A = camphor 5-monooxygenase (*camCAB*): B = *exo*-hydroxycamphor dehydrogenase (*camD*): C = 2,5-diketocamphane 1,2-monooxygenase (*camE_25-1_* + *camE_25-2_*); D = 3,6-diketo- camphane 1,6-monooxygenase *camE_36_*); E = 2-oxo-△^3^-4,5,5-trimethylcyclo-pentenylacetyl-CoA synthetase (*camF1* + *F2)*; F = 2-oxo-△^3^-4,5,5-trimethylcyclo-pentenylacetyl-CoA monooxygenase (*camG*).

Both the recorded growth substrate-dependent and growth phase-dependent changes in the titres of the inducible isoenzymic DKCMOs and their inducible flavin reductase partners Fred and PdR are indicative of elements of transcriptional control that may serve to up-regulate the pathway for the degradation of camphor in *P. putida* ATCC 17453. The extent of such controls was then investigated using the antibiotics rifampicin and actinomycin D [101]. This comprehensive study monitored the differential rates of synthesis of both chromosome-coded Fred and a number of CAM plasmid-coded activities including the ketolactonases in response to both camphor and key degradation pathway intermediates (Figure 13). Augmented by the outcomes from some relevant earlier studies [42,47,57,70], the results confirmed that the genes that code for the enantioselective DKCMOs are subject to induction by the corresponding camphor enantiomer, along with the *camRDCAB* polycistronic operon that codes for camphor 5-monooxygenase and 5-*exo*hydroxycamphor dehydrogenase. This coordinate transcriptional control of the first three successive steps of the catabolic pathway by the initial substrate represents ‘from the top’ coordinate pathway regulation, and has been reported in a number of other catabolic pathways in other *Pseudomonas* spp. [102,103]. Further, the relevant differential rates of synthesis demonstrated that each enantioselective ketolactonase as well as being induced by its own corresponding diketone substrate was cross-induced by the complementary chiral diketocamphane from the opposite enantiomeric series, and additionally back-induced by OTE. These two interesting forms of induction confirm cross-inducibility and product induction, respectively, as two additional important elements of transcriptional regulatory control of the ketolactonases in camphor-grown *P. putida* ATCC 17453.

**Figure 14:**
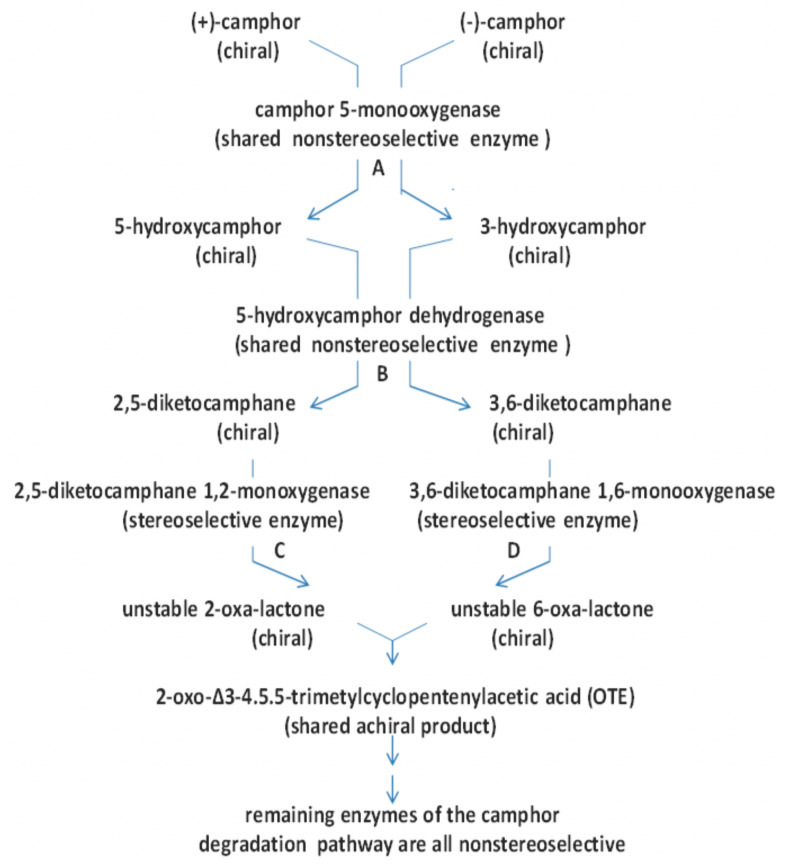
The relationships between nonselective and stereoselective enzymes that constitute the pathway for the degradation of (*rac*)-camphor to OTE in *P. putida* ATCC 17453, and the roles of chiral and achiral molecules as substrates and transcriptional regulators. A = induction by (+)-camphor and (−)-camphor, plus product induction by OTE: B = induction by (+)-camphor and (−)-camphor, plus product induction by 2,5-DKC and 3,6-DKC: C = induction by (+)-camphor, (−)-camphor and 2,5-DKC, plus cross-induction by 3,6-DKC, and product induction by OTE: D = ‘from the top’ coordinate induction by (+)-camphor, (−)-camphor, and 3,6-DKC, plus cross-induction by 2,5-DKC, and product induction by OTE: A + B + C + D = ‘from the top’ coordinate induction by (+)-camphor and (−)-camphor.

Product induction or so-called ‘from the bottom’ regulation is not unique to the DKCMOs of camphor-grown *P. putida* ATCC 17453. It has been characterised in a number of other bacterial catabolic pathways [104,105,106,107,108], and has been speculated to reflect the evolution of catabolic pathways ‘from the bottom to the top’ by the sequential acquisition of additional units of physiological function [103,104]. The confirmation of cross-inducibility of the DKCMOs by their complimentary chiral diketocamphane pathway intermediates supports earlier studies of the camphor degradation pathway which have consistently reported an equivalent significant element of cross-inducibility of both ketolactonases by each enantiomer of camphor [47,57,62,70]. The broad specificity of the relevant repressor proteins implicated by these various transcriptional controls, allied to the established patterns of coordinate induction has been suggested as a further example of a more general phenomenon characteristic of a number of different catabolic pathways of pseudomonads [35,42], the significance of which is to vest individual species with their acknowledged impressive metabolic versatility as illustrated by *P. putida* KT2440 [109]. Further light on this interesting aspect of the biochemistry of camphor-grown *P. putida* ATCC 17453 would undoubtedly result from appropriate additional in depth transcriptomic analysis [110]. These catholic patterns of transcriptional control of the isoenzymic DKCMOs contrast sharply with the established absolute stereoselectivity of the DKCMOs [31,34,57,58,62]. Possible ramifications of this need to be considered in the broader context of other known characteristics of the camphor degradation pathway to the level of OTE (Figure 2). In this respect, the known lack of stereoselectivity of both camphor 5-monooxygenase [39] and 5-*exo*hydroxycamphor dehydrogenase [111] is a significant factor, as is the unstable nature of the lactones formed by the enantiocomplementary DKCMOs which both spontaneously rearrange to the same achiral metabolite OTE. Consequently, any implied potential of *P. putida* ATCC 17453 to discriminate between (+)- and (−)-camphor as a growth substrate by selectively up-regulating the corresponding enantioselective ketolactonase (Figure 14) is negated by a combination of the established cross-inducibility of the relevant CAM plasmid genes by each of the enantiocomplementary camphor and diketocamphane isomers, and the multi-valent product induction of the genes by the shared degradation pathway intermediate OTE [101]. The very similar nucleotide sequences of the *camE_25-1_*, *camE_25-2,_*, and *camE_36_* genes on the CAM plasmid (vide supra) suggests that they probably arose by gene duplication and subsequent divergence [76], although any evolutionary advantage of the isoenzymic 2,5-DKCMOs relative to the implicit additional genetic load is difficult to imagine given the very low abundance of camphor as an available substrate in nature [26]. Considered in a broader context, a comprehensive review has concluded that enantiocomplementary and duplicate enzymes are surprisingly common in nature, and in some cases arise serendipitously [112].

**Figure 15:**
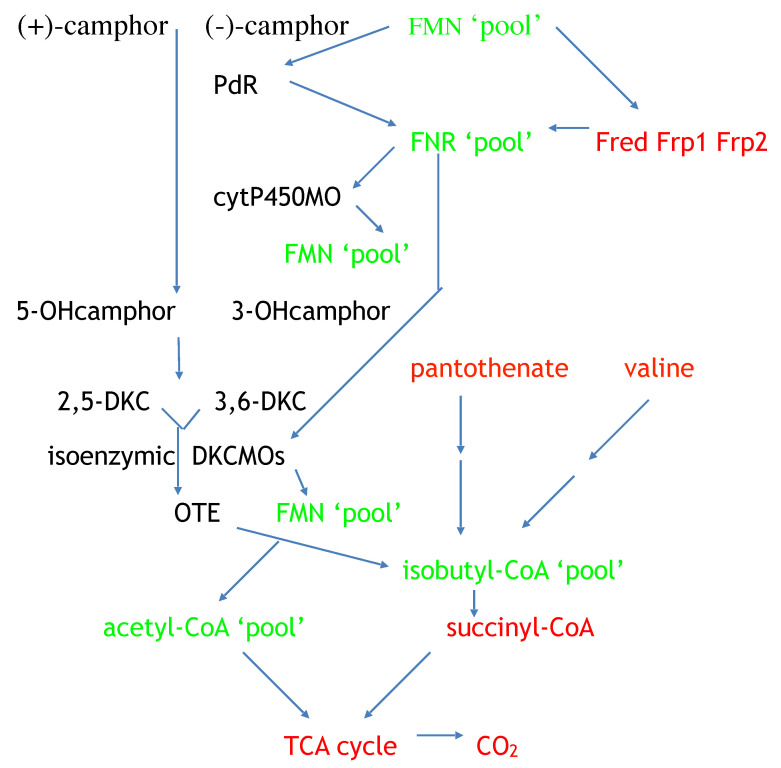
Key interrelationships between CAM plasmid-coded functions and chromosome-coded functions in (*rac*)-camphor-grown *P. putida* ATCC 17453. **black** = biomolecules exclusive to CAM plasmid-directed biochemistry: **red** = biomolecules exclusive to chromosome-directed biochemistry: **green** = biomolecules common to both plasmid-directed and chromosome-directed biochemistry. DKC = diketocamphane; FNR = FMNH_2._; cytP540MO = camphor 5-monooxygenase; OTE = 2-oxo-△^3^-4,5,5-trimethylcyclopentylacetic acid; PdR = putidaredoxin.

The most recent contribution to the sporadic six-decade history of the DKCMOs has been the confirmation for the first time that the 533 kb CAM plasmid of *P. putida* ATCC 17,453 can function as a totally autonomous extrachromosomal genetic element [8]. This was a proposal with its origins in Gunsalus’s early quest to characterise the role of oxygen-dependent enzymes in the biogeochemical carbon cycle (vide supra). His interest was fuelled further when his studies in the early 1970s [74] confirmed that an enzyme suite coded for by the CAM plasmid of *P. putida* ATCC 17453, which included four different monooxygenases, was able to catabolise C10 (+)-camphor to three molecules of C2 acetyl-CoA plus one molecule of C4 isobutyryl-CoA. Acetyl-CoA is a key metabolite that is the common end product of a significant number of universal chromosome-coded central intermediary metabolic pathways, and facilitates entry into the CO_2_-generating TCA cycle [113]. Similarly, isobutyryl-CoA is a shared intermediate with the catabolic pathways for pantothenate and valine, both chromosome-coded pathways in pseudomonads [113]. Irrespective of its metabolic origin, isobutyl-CoA can be further catabolised by chromosome-coded enzymes to the C4 TCA cycle intermediate succinyl-CoA [114]. Collectively, these activities account for every atom of C10 camphor gaining entry into the CO_2_-generating TCA cycle (Figure 15), thereby supporting Gunsalus’s metabolic autonomy proposal.

**Figure 16:**
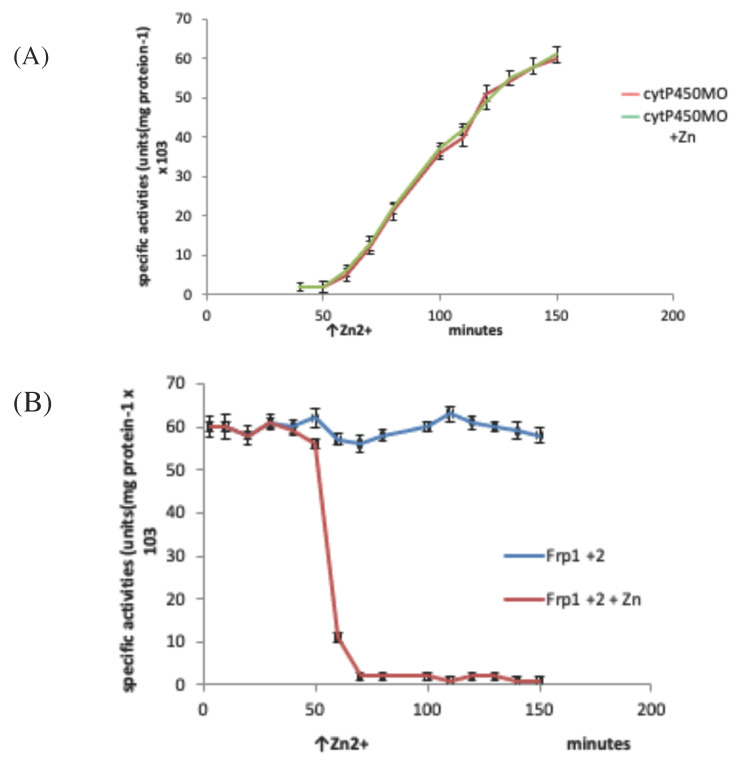
Changes in the activity of (**A**) camphor 5-monooxygenase (cytP450MO), and (**B**) the combined Frp1 + Frp2 activity in *P. putida* ATCC 17453 in the presence and absence of 30 µM Zn^2+^ added during the diauxic interlude that occurs between the swop from late log growth on succinate to early log growth on (*rac*)-camphor.

This piece of Gunsalus gospel then remained unchallenged for almost five decades until 2013 when Iwaki et al. claimed that the chromosome-coded flavin reductase Fred, was the only cognate redox flux partner for the plasmid-coded isoenzymic DKCMOs [44] which defined a dependence for the total degradation of the carboskeleton of camphor to CO_2_ on an identified chromosome-coded function. Iwaki et al.’s claimed exclusive cognate role for Fred was later proven to be incorrect when equivalent roles were confirmed for two other chromosome-coded flavin reductases (Frp1 and Frp2) and, significantly, the plasmid-coded PdR subunit of camphor 5-monooxygenase [47]. To confirm the functional significance of PdR to CAM plasmid metabolic autonomy, a strategy was developed to investigate whether DKCMO-dependent growth of *P. putida* ATCC 17453 on camphor can take place in the absence of functional activities of each of the chromosome-coded FMNH_2_-generating enzymes. Advantage was taken of the historical precedent set by the known susceptibility of microbial ferric-flavin reductases inhibition by Zn^2+^, a phenomenon first recognised, albeit not fully characterised, nearly 70 years ago [115,116,117,118]. As a consequence, relevant kinetic studies were used to establish a level of Zn^2+^ (30 µM) that completely inhibited Frp1 and Frp2 activity of *P. putida* ATCC 17453 but did not otherwise significantly affect growth of the bacterium [8]. With respect to discounting the activity of Fred, advantage was taken of the established absence of any significant detectible activity of Fred during early and mid-log trophophasic growth of the bacterium on camphor (vide supra). Additionally relevant were extensive prior studies [42,47] which confirmed that inoculation of *P. putida* ATCC 17453 into a defined medium containing both succinate and (*rac*)-camphor results in diauxic growth of the culture. Initial post-inoculation growth of the bacterium occurs exclusively at the expense of succinate, and results in the progressive depletion of succinate to below a key threshold level of 0.5 mM which then triggers a diauxic interlude. During the following 30–40 min, the remaining succinate is then further depleted to below detectible levels, and thereafter induction of the CAM plasmid-coded camphor degradation pathway enzymes is initiated. This then enables the nascent CAM plasmid-coded enzyme activities to initiate a second phase of trophophasic growth of the culture to occur exclusively at the expense of the residual (*rac*)-camphor. Significantly, at this stage of diauxic growth, chromosome-coded Fred is undetectable in cells of the bacterium, and only begins to be induced as the culture progresses into idiophasic growth [47,70]. By dividing such a diauxic culture into two then adding 30 µM Zn^2+^ to only one of the aliquots, and subsequently monitoring both aliquots concurrently throughout the following early and mid log phases of exclusively (*rac*)-camphor-dependent growth, it was possible to assess the ability of the Zn^2+^-insensitive PdR subunit of camphor 5-monooxygenase to serve as the sole functioning supplier of FMNH_2_ to the enantiocomplementary DKCMOs. Notably, the presence of Zn^2+^ resulted in no significant difference to either biomass yield, or the activity profiles of the 2,5- and 3,6-DKCMOs, and as expected for trophophasic phase cultures of (*rac*)-camphor-grown *P. putida* ATCC 17453, no detectible titres of Fred were recorded in any of the tested biomass samples [8]. Taking the titre of camphor 5-monooxygenase as reflecting that of PdR itself [94], and assaying Frp1 plus Frp2 as a combined ferric-flavin reductase activity (Figure 16), the comparative study conclusively confirmed that the recorded growth of the Zn^2+^ supplemented aliquot of the culture was achieved under conditions when the PdR subunit of camphor 5-monooxygenase was the only known functioning FMNH_2_-generating activity present in cells of the bacterium. Collectively, these outcomes indicate that, in the absence of active titres for the chromosome-coded enzymes Fred, Frp1, and Frp2, the FMNH_2_ necessary for the efficient functioning of the DKCMOs in camphor-grown *P. putida* ATCC 17453, which itself is a prerequisite for effective growth, can be supplied by CAM plasmid-coded PdR.

**Figure 17:**
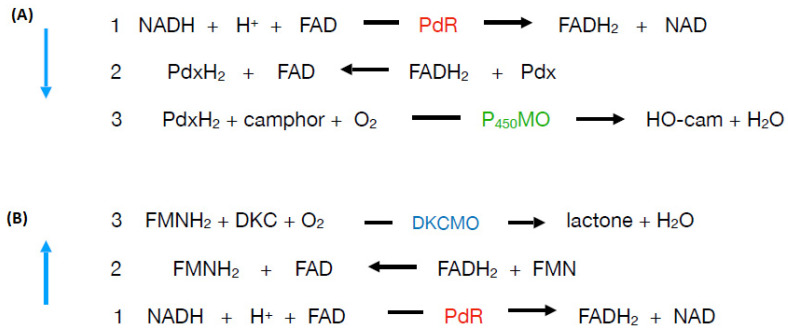
Schematic of the two contrasting roles of putidaredoxin reductase (PdR) in camphor-grown *P. putida* ATCC 17453. (**A**). cytP450MO (camphor 5-monooxygenase): Pdx (putidaredoxin) (**B**). 2,5-DKCMO (2,5-diketocamphane 1,2-monooxygenase): DKC (2,5-diketocamphane).

The intriguing question of how PdR is able to simultaneously serve an equivalent auxiliary role for two very different monooxygenase-catalysed steps in the camphor degradation pathway of *P. putida* ATCC 17453 (Figure 17) currently remains unresolved. Interestingly, a study conducted nearly 30 years ago suggested that PdR is a bifunctional enzyme that may be able to act as a NADH-dependent ferrodoxin reductase [119], while more recently the proven ability of PdR to act as an NAD(H)-dependent dithiol/disulphide oxidoreductase [120] has substantially strengthened the case for the enzyme being multi-functional. Clearly, the extent of the catalytic versatility of PdR is something that needs to be more thoroughly investigated. The outcomes from this split culture protocol do, however, finally confirm for the first time the proposal made by nearly 50 years ago by Gunsalus [74] that the CAM plasmid of *P. putida* ATCC 17453 functions as a metabolically autonomous unit able to ensure entry of all ten carbon atoms of camphor, a peripheral organic compound, into the chromosome-coded central pathways of metabolism via 3 × C2 acetyl-CoA plus 1 × C4 succinyl-CoA.

Considered from a different perspective, one possible implication of this and other related previous studies [47,70] is that in the absence of membrane-bound subcellular organelles in the prokaryotic cells of camphor-grown *P. putida* ATCC 17453, PdR and the various chromosome-coded reductases may, as circumstances dictate, contribute to a ‘pool’ of unbound FMNH_2_ (Figure 14), which is then available as a shared resource to be exploited on an ad hoc basis by the DKCMOs and any other coincidentally present FMNH_2-_dependent enzymes. This resonates directly with a speculative suggestion made by Gunsalus nearly 60 years ago [38], and which has been given additional contemporary support by the very recent confirmed ability of reductase-free biomimetic FMNH_2-_generating systems to serve as the redox partner to promote efficient 2,5-DKCMO-catalysed lactonization reactions [93]. Ironically, this pageant of events provides a paradigm of the six-decade quest to characterise the isoenzymic DKCMOs of camphor-grown *P. putida* ATCC 17453. Although not referred to as such because the concept did not exist at the time, Gunsalus’s original rather flippant 1965 suggestion of a ‘pool’ of unbound FMNH_2_ effectively delineated the DKCMOs as being what would currently be termed fd-TCMOs. However, while there is no recorded evidence that he made any further reference to his suggested ‘pool’ of unbound FMNH_2_, he did within weeks emerge as the leading proponent for the DKCMOs being true flavoproteins containing bound FMN in the active sight [36]. It was then only after a hiatus of almost half a century that the true status of the isoenzymic DKCMOs as FMNH_2_-dependent TCMOs was finally confirmed [44].

Therefore: even to this day, the episodic history of the isoenzymic DKCMOs induced by the growth of *Pseudomonas putida* ATCC 17453 on camphor, a plant natural product present in the biosphere, remains an incomplete narrative. This particular journey of scientific discovery commenced with Gunsalus’s quest to resolve the mystery of how the bicyclic terpene can interact with diatomic oxygen to thereby become integrated into the biogeochemical carbon cycle. Now, over 60 years later, it has progressed sufficiently to validate another of Gunsalus’s astute predictions by confirming that the CAM plasmid of *P putida* ATCC 17453 can function as a totally autonomous metabolic entity. However, the journey is not yet over and the historical epilogue remains incomplete, as illustrated by the current unresolved mystery of how PdR can efficiently serve two distinctly different roles concurrently in the camphor-grown bacterium. As evidenced by this review, the path the journey has taken throughout those intervening six decades has been determined be a veritable potpourri of both positive and negative influences, but that is so often a defining characteristic of the search for the transitional truth of knowledge.

## Data Availability

Data supporting reported results can be found in the relevant cited publications.

## References

[1] Heine T., van Berkel W.J.H., Gassner G., van Pee K.-H., Tischler D. (2018). Two-component FAD-dependent. monooxygenases: Current knowledge and biotechnological opportunities. Biology.

[2] Valton J., Filisetti L., Montecave M., Niviere V. (2004). A two-component flavin-dependent monooxygenase involved in actinorhodin biosynthesis in *Streptomyces coelicolor*. J. Biol. Chem..

[3] Tu S.C. (2001). Reduced flavin: Donor acceptor enzymes and mechanisms of chanelling. Antiox. Redox Signal..

[4] Suckaritakul J., Tinikul R., Chaiyen P. (2014). Mechanisms of reduced flavin transfer in the two-component flavin-dependent monooxygenases. Arch. Biochem. Biophys..

[5] Warburg O., Christian W. (1933). Uber das gelbe Ferment und seine Wirkungen. Biochem. Z..

[6] Matthews R.G., Massey V. (1969). Isolation of Old Yellow Enzyme in free and complexed forms. J. Biol. Chem..

[7] Elegheert J., Beeuman A.B.J., Savvides S. (2017). Structural dissection of *Shewanella oneidensis* old yellow enzyme 4 bound to a Meisenheimer complex and (nitro)phenolic ligands. FEBS Lett..

[8] Willetts A. (2019). Conferring the metabolic self-sufficiency of the CAM plasmid of *Pseudomonas putida* ATCC 17453. The key role of putedaredoxn reductase. Microorganisms.

[9] Conrad H.E., Corey E.J., Gunsalus I.C., Hartmann R. (1961). Enzymatic lactonization of terpenoids. Fed. Proc..

[10] Pray L.A. (2008). Discovery of DNA structure and function: Watson and Crick. Nat. Educ..

[11] Wieland H. (1931). Neuere untersuchungen über die biologische oxydation. Angew. Chem..

[12] Kotake Y., Masayama I. (1936). The intermediary metabolism of tryptophan. XVIII. The mechanism of formation of kynurenine from tryptophan. Z. Physiol. Chem..

[13] Turfitt G.E. (1948). The microbial degradation of steroids: 4. Fission of the steroid molecule. Biochem. J..

[14] Kramli A., Horvath J. (1948). Microbial oxidation of sterols. Nature.

[15] Fried J., Thoma R.W., Gerke J.R., Herz J.E., Dinon M.N., Partmen D. (1952). Oxidation of steroids by microorganisms.II. Hydroxylation at position 1 and synthesis of cortisone from Reichstein’s compound S. J. Am. Chem. Soc..

[16] Peterson D.H., Nathan A.H., Meister P.D., Eppstein S.H., Murray H.C., Weintraub A., Reineke L.M., Leigh H.M. (1953). Microbial Transformation of steroids.VI. Preparation of 11⍺-hydroxy-6-dehydroprgesterone. J. Am. Chem. Soc..

[17] Fried J., Thoma R.W., Klingberg A. (1953). Oxidation of steroids by microorganisms.III. Side chain degradation, ring D cleavage, and dehydrogenation of the A ring. J. Am. Chem. Soc..

[18] Peterson D.H., Eppstein S.H., Meister P., Murray H.C., Leigh H.M., Weintraub A., Reineke L.M. (1953). Microbial Transformation of steroids. IX. Degradation of C21 steroids to C19 lactones and testololactone. J. Am. Chem. Soc..

[19] Mason H.S., Fowlks W.L., Peterson E. (1955). Oxygen transfer and electron transport by phenolase complex. J. Am. Chem. Soc..

[20] Mason H.S. (1955). Comparative biochemistry of the phenolase complex. Adv. Enzymol..

[21] Hayaishi O., Katagiri M., Rothberg S. (1955). Mechanism of the pyrocatechase reaction. J. Am. Chem. Soc..

[22] Hayaishi O., Katagiri M., Rothberg S. (1957). Studies on oxygenases: Pyrocatechase. J. Biol. Chem..

[23] Klingenberg M. (1958). Pigments of rat liver microsomes. Archiv. Biochem. Biophys..

[24] Garkinkel D. (1958). Studies on pig liver microsomes. 1. Enzymic and pigment composition of different microsomal fractions. Arch. Biochem. Biophys..

[25] Tanaka T., Knox W.E. (1959). The nature and mechanism of the tryptophan pyrrolase (peroxidase-oxidase) reaction of *Pseudomonas* and rat liver. J. Biol. Chem..

[26] Trudgill P.W., Sokatch J.R. (1986). Terpenoid metabolism in *Pseudomonas*. The Bacteria.

[27] O’Kane D.J., Gunsalus I.C. (1947). Acessory factor requirement for pyruvate oxidation. J. Bacteriol..

[28] O’Kane D.J., Gunsalus I.C. (1948). Pyruvic acid metabolism. A factor required for oxidation by *Streptomyces faecalis*. J. Bacteriol..

[29] Bradshaw W.H., Conrad H.E., Corey E.J., Gunsalus I.C., Lednicer D. (1959). Microbial degradation of (+)–camphor. J. Am. Chem. Soc..

[30] Conrad H.E., DuBus R., Gunsalus I.C. (1961). An enzyme system for cyclic lactonization. Biochem. Biophys. Res. Commun..

[31] Willetts A. (1998). Structural studies and synthetic applications of Baeyer-Villiger monooxygenases. Trends Biotechnol..

[32] Bertland A.U., Johnson S., Gunsalus I.C. (1963). Induced enzymes in terpene metabolism: An FMN-coupled DPNH oxidase in pseudomonads. Bacteriol. Proc..

[33] LeGall J., Gunsalus I.C. (1963). Growth of terpene oxidizing pseudomonads. Bacteriol. Proc..

[34] Jones K.H., Smith R.T., Trudgill P.W. (1993). Diketocamphane enantiomer- specific ‘Baeyer-Villiger’ monooxygenases from camphor-grown *Pseudomonas putida* ATCC 17453. J. Gen. Microbiol..

[35] Gunsalus I.C., Trudgill P.W., Cushman D., Conrad H.E. (1965). Stereospecific biological oxygenation of terpenoids. Symposium on Recent Advances in the Chemistry of Terpenoids.

[36] Conrad H.E., DuBus R., Namtvedt M.J., Gunsalus I.C. (1965). Mixed function oxidation. II. Separation and properties of the enzymes catalysing camphor lactonization. J. Biol. Chem..

[37] Conrad H.E., Lieb K., Gunsalus I.C. (1965). Mixed function oxidation. III. An electron transport complex in camphor ketolactonization. J. Biol. Chem..

[38] Gunsalus I.C., Conrad H.E., Trudgill P.W., King T.E., Mason H.S., Morrish M. (1965). Generation of active oxygen for mixed function oxidation. Oxidases and Related Redox Systems.

[39] Hedegaard J., Gunsalus I.C. (1965). Mixed function oxidation. IV. An induced methylene hydroxylase in camphor oxidation. J. Biol. Chem..

[40] Trudgill P.W., DuBus R., Gunsalus I.C. (1966). Mixed function oxidation. V. Flavin interaction with a reduced diphosphopyridine nucleotide dehydrogenase, one of the enzymes participating in camphor lactonization. J. Biol. Chem..

[41] Trudgill P.W., DuBus R., Gunsalus I.C. (1966). Mixed function oxidation. VI. Purification of a tightly coupled electron transport complex in camphor lactonization. J. Biol. Chem..

[42] Gunsalus I.C., Bertland A.U., Jacobson L.A. (1967). Enzyme induction and repression in anabolic and catabolic pathways. Arch. Mikrobiol..

[43] Yu C.A., Gunsalus I.C. (1969). Monooxygenases. VII. Camphor lactonase I and the role of three protein components. J. Biol. Chem..

[44] Iwaki H., Grosse S., Bergeron H., Leisch H., Morley K., Hasegawa Y., Lau P.C. (2013). Camphor pathway redox: Functional recombinant expression of 2,5- and 3,6-diketocam … phane monooxygenases in *Pseudomonas putida* ATCC 17453 with their cognate flavin reductase catalysing Baeyer-Villiger reactions. Appl. Environ. Microbiol..

[45] Shapiro A.L., Vinuela E., Maizel J.V. (1967). Molecular weight estimation of polypeptide chains by electrophoresis in SDS-polyacrylamide gels. Biochem. Biophys. Res. Commun..

[46] Unger B.P., Sligar S.G., Gunsalus I.C., Sokatch J.R. (1986). Pseudomonas cytochrome P-450. The Bacteria.

[47] Willetts A., Kelly D.R. (2016). Flavin-dependent redox transfers by two-component diketocamphane monooxygenases of camphor-grown *Pseudomonas putida* NCIMB 10007. Microorganisms.

[48] Gunsalus I.C., Marshall V.P. (1971). Monoterpene dissimilation. CRC Crit. Rev. Microbiol..

[49] Stanier R.Y. (1947). Simultaneous adaption: A new technique for the study of metabolic pathways. J. Bacteriol..

[50] Jacobson L.A. (1967). Enzyme Induction and Repression in the Catabolism of (+)–Camphor by *Pseudomonas putida*. Ph.D. Thesis.

[51] Poulos T.L. (2014). Heme enzyme structure and function. Chem. Rev..

[52] Laskin A.I., Lechevalier H. (1984). Microbial transformations. CRC Handbook of Microbiology.

[53] Baumberg S., Mandelstam J., McQuillen K., Dawes I. (1982). Co-ordination of metabolism. Biochemistry of Bacterial Growth.

[54] Taylor D.G., Trudgill P.W. (1986). Camphor revisited: Studies of 2,5-diketocamphane 1,2-monooxygenase from *Pseudomonas putida* ATCC 17453. J. Bacteriol..

[55] Kadow M., Sass S., Schmidt M., Bornscheuer U. (2011). Recombinant expression and purification of the 2,5-diketocamphane 1,2-monooxygenase from the camphor metabolizing *Pseudomonas putida* strain NCIMB 10007. AMB Express.

[56] Kadow M., Loschinski K., Sass S., Schmidt M., Bornscheuer U. (2012). Completing the series of BVMOs involved in camphor metabolism of *Pseudomonas putida* NCIMB 10007 by identification of the two missing genes, their functional expression in *E. coli*, and biochemical characterization. Appl. Microbiol. Biotechnol..

[57] Grogan G. (1995). Microbial biotransformations: Oxygenation of Cyclic Ketones by Baeyer- Villiger Monooxygenases from *Pseudomonas putida* NCIMB 10007. Ph.D. Thesis.

[58] Beecher J.E., Grogan G., Roberts S.M., Willetts A. (1996). Enantioselective biooxidations by the enantiocomplementary diketocamphane monooxygenase isoenzymes from *Pseudomonas putida* NCIMB 10007. Biotechnol. Lett..

[59] Beecher J.E., Willetts A. (1998). Biotransformation of organic sulphides. Predictive active site models for sulfoxidation catalysed by the 2,5-diketocamphane 1,2-monooxygenase and 3,6-diketocamphane 1,6-monooxygenase, enantiocomplementary enzymes from *Pseudomonas putida* NCIMB 10007. Tetrahedron Asymm..

[60] Isupov M.N., Schroder E., Gibson R.P., Beecher J., Donadio G., Saneei V., Dcunha S.A., McGhie E.J., Sayer C., Davenport C.F. (2018). The oxygenating constituent of 3,6-diketocamphane monooxygenase from the CAM plasmid of *Pseudomonas putida*: The first crystal structure of a type II Baeyer-Villiger monooxygenase. Corrigendum. Acta Crystalog. Sect. D.

[61] Levitt M., Newton R.F., Roberts S.M., Willetts A. (1990). Preparation of optically active 6’-fluorocarbocyclic nucleosides utilizing an enantiospecific enzyme-catalysed Baeyer–Villiger type oxidation. J. Chem. Soc. Chem. Commun..

[62] Gagnon R., Grogan G., Roberts S.M., Villa R., Willetts A. (1995). Enzymatic Baeyer-Villiger oxidation of some bicyclo[2.2. 1]heptan-2-ones using monooxygenases from *Pseudomonas putida* NCIMB 10007: Enantioselective preparation of a precursor of azadirachtin. J. Chem. Soc. Perkin Trans..

[63] Adger B., Bes T., Grogan G., McCague R., Pedragosa-Moreau S., Roberts S.M., Villa R., Wan P.H., Willetts A. (1995). Applications of enzymic Baeyer-Villiger oxidations of 2-substituted cycloalkanones to the total synthesis of *R*-(+)−lipoic acid. J. Chem. Soc. Chem. Commun..

[64] Willetts A. (2019). Characterised flavin-dependent two-component monooxygenases from the CAM plasmid of *Pseudomonas putida* ATCC 17453 (NCIMB 10007): Ketolactonases by another name. Microorganisms.

[65] Meighen E.A. (1991). Molecular biology of bacterial bioluminescence. Microbiol. Rev..

[66] Kadow M. (2012). Baeyer-Villiger Monooxygenases Involved in Camphor Degradation. Ph.D. Thesis.

[67] Balke K., Kadow M., Malin H., Sag S., Bornscheuer U.T. (2012). Discovery, application, and protein engineering of Baeyer-Villiger monooxygenases for organic synthesis. Org. Biomol. Chem..

[68] Fontecave M., Eliasson R., Reichard P. (1987). NAD(P)H-flavin oxidoreductase of *Escherichia coli*: A ferric iron reductase participating in the generation of the free radical of ribonucleotide reductase. J. Biol. Chem..

[69] Leipold F., Wardenga R., Bornscheuer U.T. (2012). Cloning, expression and characterization of a eukaryotic cycloalkanone monooxygenase from *Cylindrocarpon radicicola* ATCC 11011. Appl. Microbiol. Biotechnol..

[70] Willetts A., Kelly D.R. (2014). Multiple native flavin reductases in camphor-metabolising *Pseudomonas putida* NCIMB 10007: Functional interaction with two-component diketocamphane monooxygenase isoenzymes. Microbiology.

[71] Russell T.R., Tu S.C. (2004). *Aminobacter aminovorans* NADH:flavin oxidoreductase His 140: A highly conserved residue critical for NADH binding and utilization. Biochemistry.

[72] Imagawa T., Tsurumura T., Sugimoto Y., Aki K., Ishidoh K., Kuramitsuu S., Tsuge H. (2011). Structural basis of free reduced flavin generation by flavin reductase from *Thermus thermophilus* HB8. J. Biol. Chem..

[73] Knobel H.R., Egli T., van der Meer J.R. (1996). The cloning and characterization of the genes encoding nitrilotriacetate monooxygenase of *Chelatobacter heintzii* ATCC 29600. J. Bacteriol..

[74] Rheinwald J.G., Chakrabarty A.M., Gunsalus I.C. (1973). A transmissible plasmid controlling camphor oxidation in *Pseudomonas putida*. Proc. Natl. Acad. Sci. USA.

[75] Frantz B., Chakrabarty A.M., Sketch J.R. (1986). Degradartive plasmids in *Pseudomonas*. The Bacteria.

[76] Ohno S. (1970). Evolution by Gene Duplication.

[77] Packer M.S., Liu D.R. (2015). Methods for the directed evolution of proteins. Nature Rev. Genet..

[78] Jones J.B., Jakovac I.J. (1982). A new cubic-space section model for predicting the specificity of horse liver alcohol dehydrogenase-catalysed oxidoreductions. Can. J. Chem..

[79] Henzler-Wildman K., Kern D. (2007). Dynamic personalities of proteins. Nature.

[80] Poulos T.L., Finzel B.C., Howard A.J. (1987). High-resolution crystal structure of cytochrome P450_cam_. J. Mol. Biol..

[81] McGhie E.J. (1998). Studies on Monooxygenases from the Camphor Degradation Pathway in *Pseudomonas putida* NCIMB 10007 that are Important in the Catalysis of Baeyer-Villiger Biotransformation Reactions. Ph.D. Thesis.

[82] McGhie E.J., Isupov M.N., Schroder E., Littlechild J.A. (1998). Crystallization and preliminary X-ray diffraction studies of the oxygenating subunit of 3,6-diketocamphane monooxygenase from *Pseudomonas putida*. Acta Crystalogr. Sect. D..

[83] Isupov M.N., Lebedev A.A. (2008). NCS-constrained exhaustive search using oligomeric models. Acta Crystalogr. Sect. D.

[84] Fisher A.J., Thompson T.B., Thoden J.B., Baldwin T.O., Rayment I. (1996). The 1.5-Å resolution crystal structure of bacterial luciferase in low salt conditions. J. Biol. Chem..

[85] Rost B. (1999). Twilight zone of protein sequence alignment. Protein Eng..

[86] Campbell Z.T., Weichsel S., Montfort W.R., Baldwin T.D. (2009). Crystal structure of the bacterial luciferase/flavin complex provides insight into the function of the β subunit. Biochemistry.

[87] Willetts A. (2017). Reply to the comment by Littlechild and Isupov. Microorganisms.

[88] Mϋller F., Hemmerich P., Ehrenberg A., Kamin H. (1971). On the molecular and submolecular structure of flavin free radicals and their properties. Flavins and Flavoproteins.

[89] Mirza I.A., Yachnin B.J., Wang S., Grosse S., Bergeron H., Imura A., Berhuis A.M. (2009). Crystal structures of cyclohexanone monooxygenase reveal complex domain movements and a sliding cofactor. J. Am. Chem. Soc..

[90] Chakrabarty A.M., Gunsalus C.F., Gunsalus I.C. (1968). Transduction and the clustering of genes in fluorescent Pseudomonads. Proc. Natl. Acad. Sci. USA.

[91] Yeom J., Jeon C.O., Madsen E.L., Park W. (2009). Ferrodoxin-NADP reductase from *Pseudomonas putida* functions as a ferric reductase. J. Bacteriol..

[92] Peterson J.A., Lorence M.C., Amarneh B. (1990). Putidaredoxin reductase and putidaredoxin: Cloning, sequence determination and heterologous expression of the proteins. J. Biol. Chem..

[93] Rollig R., Paul C.E., Claeys-Bruno M., Duquesne K., Kara S., Alphand V. (2021). Divorce in the two-component BVMO family: The single oxygenase for enantioselective chemo-enzymatic Baeyer-Villiger oxidations. Org. Biomed. Chem..

[94] Tyson C.A., Lipscomb J.A., Gunsalus I.C. (1972). The roles of putidaredoxin and P450cam in methylene hydroxylation. J. Biol. Chem..

[95] Vising L.C. (1992). Roles of secondary metabolism in microbes. Ciba Found. Symp..

[96] Loescheke A., Thies S. (2015). *Pseudomonas putida*—A versatile host for the production of natural products. Appl. Microbiol. Biotechnol..

[97] Gross H., Loper J.E. (2009). Genomics of secondary metabolite production by *Pseudomonas* spp.. Nat. Prod. Rep..

[98] Thibaut D., Ratet N., Bisch D., Faucher D., Debussch L., Blanche F. (1995). Purification of the two-enzyme system catalysing the oxidation of the D-proline residue of pristinamycin IIB during the last step of pristinamycin IIA biosynthesis. J. Bacteriol..

[99] Parry R.J., Li W. (1997). An NADPH:FAD oxidoreductase from the valinimycin producer, *Streptomyces viridifaciens*. J. Biol. Chem..

[100] Valton J., Mathevon C., Fontecave M., Niviere V., Ballou D.P. (2008). Mechanism and regulation of the two-component FMN-dependent monooxygenase ActVA-ActVB from *Streptomyces coelicolor*. J. Biol. Chem..

[101] Willetts A., Masters P., Steadman C. (2018). Regulation of camphor metabolism: Induction and repression of relevant monooxygenases in *Pseudomonas putida* NCIMB 10007. Microorganisms.

[102] Ornston L.N. (1971). Regulation of catabolic pathways in *Pseudomonas*. Bacteriol. Rev..

[103] Ornston L.N., Parke D. (1977). The evolution of induction mechanisms in bacteria: Insights derived from the study of the β-ketoadipate pathway. Curr. Top. Cell Regul..

[104] Palleroni N.J., Stanier R.Y. (1964). Regulatory mechanisms governing synthesis of the enzymes for tryptophan oxidation in *Pseudomonas fluorescens*. J. Gen. Microbiol..

[105] Wheelis M.L., Stanier R.Y. (1970). The genetic control of dissimilatory pathways in *Pseudomonas putida*. Genetics.

[106] Newell C.P., Lessie T.G. (1970). Induction of histidine-degrading enzymes in *Pseudomonas aeruginosa*. J. Bacteriol..

[107] Leidigh B.J., Wheelis M.L. (1973). Genetic control of the histidine dissimilatory pathway in *Pseudomonas putida*. Mol. Gen. Genet..

[108] Parada J.L., Magasanik B. (1975). Expression of the hut operon of *Salmonella typhimurium* in *Klebsiella aerogenes* and *Escherichia coli*. J. Bacteriol..

[109] Nelson K.E., Weinel C., Paulsen I.T., Dodson R.J., Hilbert H., Martins dos Santos V.A.P., Fouts D.E., Gill S.R., Pop M., Holmes M. (2002). Complete genome sequence and comparative analysis of the metabolically versatile *Pseudomonas putida* KT2440. Environ. Microbiol..

[110] Lowe R., Shirley N., Bleackley M., Dolan S., Shafee T. (2017). Transcriptomic technologies. PLOS Comput. Biol..

[111] Hartline R.A., Gunsalus I.C. (1971). Induction specificity and catabolite repression of the early enzymes in camphor degradation by *Pseudomonas putida*. J. Bacteriol..

[112] Mugford P.F., Wagner U.G., Jiang Y., Faber K., Kazlauskas R.J. (2008). Enantiocomplementary enzymes: Classification, molecular basis for their enantiopreference, and prospects for mirror-image biotransformations. Angew. Chem. Int. Ed..

[113] Dagley S., Nicholson D.E. (1970). An Introduction to Metabolic Pathways.

[114] Massey L.K., Sokatch J.R., Conrad R.S. (1976). Branched-chain amino acid catabolism in bacteria. Bacteriol. Rev..

[115] Baghdianz A. (1952). Role du zinc sur l’apparition de la composante du ’pigment’ *de Pseudomonas fluorescens* (Flugge-Migula). Arch. Sci..

[116] Chakrabarty A.M., Roy S.C. (1964). Effect of trace elements on the production of pigments by a pseudomonad. Biochem. J..

[117] Huyer M., Page W.J. (1988). Zn^2+^ increases siderophore production in *Azotobacter vinelandii*. Appl. Environ. Microbiol..

[118] Huyer M., Page W.J. (1989). Ferric reductase activity in *Azotobacter vinelandii* and its inhibition by Zn^2+^. J. Bacteriol..

[119] Jungermann K., Trauer R.K., Leimenstoff G., Decker K. (1993). Function of reduced pyridine nucleotide-ferrodoxin oxidoreductases. Biochem. Biophys. Acta Bioenerg..

[120] Sevroiukova I.F., Poulos T.L. (2002). Putidaredoxin reductase, a new function for an old protein. J. Biol. Chem..

